# Strategies for the production of biochemicals in bioenergy crops

**DOI:** 10.1186/s13068-020-01707-x

**Published:** 2020-04-15

**Authors:** Chien-Yuan Lin, Aymerick Eudes

**Affiliations:** 1 Joint BioEnergy Institute, Emeryville, CA 94608 USA; 2 Environmental Genomics and Systems Biology Division, Lawrence Berkeley National Laboratory, Berkeley, CA 94720 USA

**Keywords:** Bioenergy crops, Shikimate, Isoprenoids, Terpenes, Metabolic engineering

## Abstract

Industrial crops are grown to produce goods for manufacturing. Rather than food and feed, they supply raw materials for making biofuels, pharmaceuticals, and specialty chemicals, as well as feedstocks for fabricating fiber, biopolymer, and construction materials. Therefore, such crops offer the potential to reduce our dependency on petrochemicals that currently serve as building blocks for manufacturing the majority of our industrial and consumer products. In this review, we are providing examples of metabolites synthesized in plants that can be used as bio-based platform chemicals for partial replacement of their petroleum-derived counterparts. Plant metabolic engineering approaches aiming at increasing the content of these metabolites in biomass are presented. In particular, we emphasize on recent advances in the manipulation of the shikimate and isoprenoid biosynthetic pathways, both of which being the source of multiple valuable compounds. Implementing and optimizing engineered metabolic pathways for accumulation of coproducts in bioenergy crops may represent a valuable option for enhancing the commercial value of biomass and attaining sustainable lignocellulosic biorefineries.

## Background

Bioenergy crops are grown with low inputs to generate lignocellulosic biomass that constitutes a sustainable source of renewable energy. One appealing purpose of bioenergy crops is the deconstruction of their biomass into aromatics and simple sugars for downstream conversion into bioproducts. This strategy has been put forward for the environmentally sustainable production of fuels and chemicals currently derived from petroleum refining [1]. However, several challenges still need to be overcome to render bio-based products cost-competitive vis-à-vis their petroleum-based counterparts. For biological conversion of plant biomass hydrolysates, robust engineered microbial strains are required to allow efficient production of the desired chemicals at high yields, titers, and rates using complex mixtures of low-molecular weight monomers as substrates. Moreover, achieving higher plant biomass yields at reduced cost and enabling efficient deconstruction of the recalcitrant lignocellulosic material represent two other important milestones towards the cost-effectiveness of biochemical production [2, 3].

Plant genetic engineering has the potential to overcome some of these challenges. For example, bioenergy crops can be genetically improved for producing more biomass, being more resilient to stresses like pathogens and drought conditions, or synthesizing cell wall materials with reduced recalcitrance towards deconstruction processes [4, 5]. In addition, metabolic engineering offers the possibility to increase the value of biomass by overproducing in planta a wide range of products [6, 7]. These valuable chemicals can be naturally produced by certain plants, but are often present in too low quantities in dedicated bioenergy crop for their exploitation. In some cases, the target chemicals do not occur in plants and implementation of de-novo metabolic pathways is required for their synthesis. Biochemicals isolated from engineered energy crops can be further modified biologically or catalytically to serve several industrial sectors. In particular, in the scope of a bio-based approach for producing large quantities of commodity chemicals to satisfy global markets, the use of bioenergy crops as green factories may represent a valuable option since these are expected to be grown on large acreage of land, including marginal lands not suitable for food crop cultivation. In fact, accumulation of bioproducts or metabolic intermediates in crops may benefit specific biorefinery configurations in which hydrolysates are fractionated to derive maximum value from each component [2]. For example, it has been recently documented from techno-economic analyses that co-production of high-value chemicals in bioenergy crops has the potential to ameliorate the economics of advanced biofuels obtained from lignocellulosic biomass [8].

**Fig. 1:**
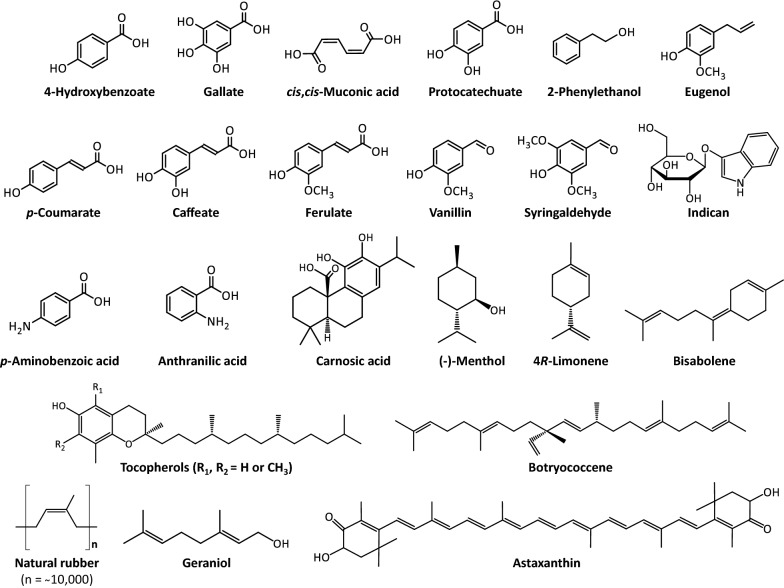
Structures of the chemicals of interest described in this review

In this review, we are inventorying metabolic routes that constitute or originate from the shikimate and isoprenoid pathways in plants. Especially, we summarize engineering approaches leading to the overproduction of several chemicals of interest derived from these two pathways (Fig. 1). In many instances, we illustrate how exploiting metabolic steps or enzymes found in non-plant organisms enable the production of specific biochemicals de-novo or at higher levels. Various potential or already existing industrial applications for these plant-based chemicals are presented.

## Biochemicals derived from the shikimate pathway

**Fig. 2:**
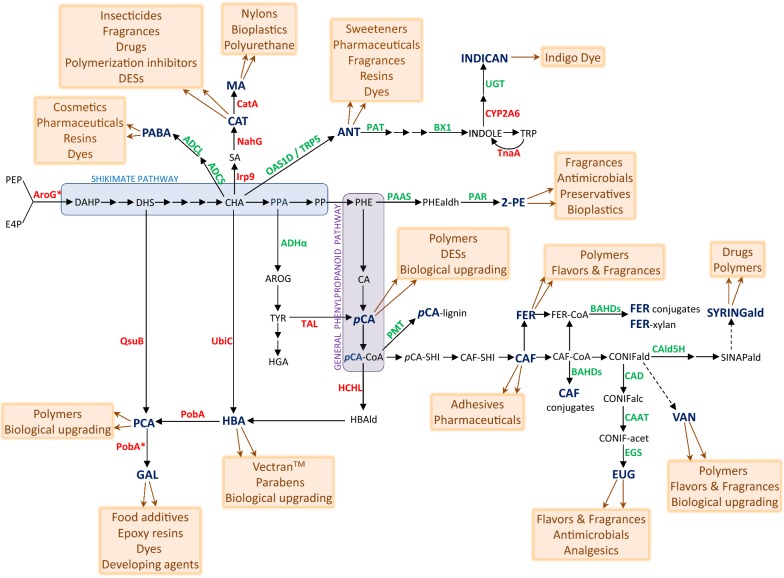
Proposed metabolic steps for the synthesis of chemicals derived from the shikimate and general phenylpropanoid pathways. Enzyme names are indicated in the case of steps that have been the object of metabolic engineering. Green and red fonts are used to denote enzymes from plant and non-plant origins, respectively. Asterisks indicate that a mutant version of the enzyme was used. ADCL, 4-amino-4-deoxychorismate-lyase; ADCS, 4-amino-4-deoxychorismate synthase; ADHα, arogenate dehydrogenase alpha; ANT, anthranilate; AroG, 3-Deoxy-D-arabino-heptulosonate 7-phosphate (DAHP) synthase; AROG, arogenate; BAHDs, BAHD transferases; BX1, indole synthase; CAAT, coniferyl alcohol:acetyl-CoA transferase; CAD, cinnamyl alcohol dehydrogenase; CAF-CoA, caffeoyl-CoA; CAF, caffeate; CAld5H, coniferladehyde 5-hydroxylase; CatA, catechol 1,2-dioxygenase; CAT, catechol; CA, cinnamate; CHA, chorismate; CONIFalc, coniferyl alcohol; CONIFald, coniferaldehyde; CONIF-acet, coniferyl acetate; CYP2A6, cytochrome P450 monooxygenase; DAHP, 3-Deoxy-d-arabino-heptulosonate 7-phosphate; DES, deep eutectic solvents; DHS, 3-dehydroshikimate; EGS, eugenol synthase; EUG, eugenol; E4P, erythrose 4-phosphate; FER-CoA, feruloyl-CoA; FER, ferulate; GAL, gallate; Hbald, 4-hydroxybenzaldehyde; HBA, 4-hydroxybenzoate; HCHL, hydroxycinnamoyl-CoA hydratase-lyase; HGA, homogentisate; Irp9, salicylate synthase; MA, muconic acid; NahG, salicylate hydroxylase; OAS1D, rice anthranilate synthase α-subunit; PAAS, phenylacetaldehyde synthase; PABA, *p*-aminobenzoate; PAR, phenylacetaldehyde reductase; PAT, phosphoribosyl transferase; *p*CA-CoA, *p*-coumaroyl-CoA; *p*CA-SHI, *p*-coumaroyl-shikimate; *p*CA, *p*-coumarate; PCA, protocatechuate; PEP, phosphoenolpyruvate; PHEald, phenylacetaldehyde; PHE, phenylalanine; PMT; *p*-coumaroyl-CoA:monolignol transferase, PobA, 4-hydroxybenzoate 3-monooxygenase; PPA, prephenate; PP, phenylpyruvate; QsuB, 3-dehydroshikimate dehydratase; SA, salicylate; SINAPald, sinapaldehyde; SYRINGald, syringaldehyde; TAL, tyrosine ammonia-lyase; TnaA, tryptophanase; TRP, tryptophan; TRP5, Arabidopsis feedback-insensitive anthranilate synthase α-subunit; TYR, tyrosine; UbiC, chorismite pyruvate-lyase; UGT, indoxyl glucosyltransferase; VAN, vanillin; 2-PE, 2-phenylethanol

The shikimate pathway, which is confined to plastids in plants, is responsible for the synthesis of aromatic amino acids that are precursors to secondary metabolites such as pigments, alkaloids, hormones, and phenylpropanoids including lignin [9]. In microbes, the shikimate pathway has been exploited for the production of aromatic chemicals which are otherwise derived from petroleum-based benzene, toluene and xylene [10, 11]. Nevertheless, most aromatic compounds used for industrial applications are still synthesized chemically due to the inefficiency of current biological production methods. Notably, several metabolic steps from these engineering approaches developed in microbial systems have been successfully implemented in plants, which open new avenues for the production of shikimate-derived metabolites in bioenergy crops (Fig. 2). Several metabolites derived from the shikimate and phenylpropanoid pathways find applications in medicine [12], and other emerging applications for these chemicals include the manufacturing of biopolymers [13]. In particular, because of their aromatic nature, intermediates of the shikimate pathway have the potential to generate bio-replacements for commonly fossil fuel-derived aromatics.

### 4-Hydroxybenzoic acid

4-Hydroxybenzoic acid (4-HBA) is synthesized from benzene on industrial scale for the synthesis of liquid crystal polymers used to manufacture fibers such as Vectran™. Strategies have been explored for converting 4-HBA into the commodity chemical terephthalic acid, a precursor to the polyester polyethylene terephthalate (PET) used to make clothing and plastic bottles [14]. 4-HBA is also the precursor for parabens, which are preservatives used in cosmetic and pharmaceutical products [15–17]. Considering that 4-HBA is produced by plants and is potentially released from biomass during pretreatment processes, its biological upgrading represents a conceivable option towards valorization [18]. Microbial strains with the capacity to catabolize 4-HBA are therefore appropriate chassis for biological upgrading. For example, certain oleaginous bacteria from the *Rhodococcus* genus use 4-HBA as sole carbon source for the production of triacylglycerols that can be transesterified into fatty acid methyl esters for biodiesel applications [19]. Similarly, the betaproteobacterium *Pandoraea* sp. ISTKB was shown to use 4-HBA for growth and production of polyhydroxyalkanoate biopolyesters [20]. Other examples include the biological funneling of 4-HBA into valuable catabolic pathway intermediates such as muconic acid, 2-pyrone-4,6-dicarboxylic acid, beta-ketoadipic acid, and isocinchomeronic acid using engineered strains of *Novosphingobium aromaticivorans* and *Pseudomonas putida* [21–23].

Overproduction of 4-HBA has been achieved in plants by overexpression of bacterial chorismate pyruvate-lyase (UbiC) or hydroxycinnamoyl-CoA hydratase-lyase (HCHL) (Fig. 2) [24–27]. In these cases, the UbiC enzyme is fused to the sequence of a chloroplast transit peptide to reroute the chorismate pool generated from the shikimate pathway [24], whereas native HCHL converts *p*-coumaroyl-CoA into 4-hydroxybenzaldehyde which becomes oxidized by endogenous dehydrogenase(s) into 4-HBA [25, 27]. For both strategies, 4-HBA accumulates as glucose-conjugated forms, presumably stored in vacuoles, suggesting an export of 4-HBA from chloroplasts to the cytosol in the case of the plastid-targeted UbiC approach. 4-HBA glucosides are readily extracted from plant biomass using aqueous methanol solvents [24–27]. Assessment of both strategies in sugarcane showed that HCHL was more efficient than UbiC, resulting in the production of 7.3% and 1.5% dry weight (DW) of 4-HBA glucosides in leaves and stems, respectively [26]. Moreover, analysis of lignin purified from Arabidopsis plants transformed with a HCHL gene showed the presence of 4-HBA and syringaldehyde units, as well as a reduction in the lignin degree of polymerization, which results in increases of biomass saccharification efficiency [27, 28]. Therefore, the HCHL engineering strategy has the potential to bring two valuable traits in bioenergy crops, namely ‘enhanced biomass deconstructability’ and ‘value-added coproduct’. Lignin in cell walls could represent a preferred site for 4-HBA accumulation in case large amount of intracellular 4-HBA leads to toxic effects. Although several plants such as poplar, aspen, willow, and certain palm species contain 4-HBA esters in their lignins, the exact mechanism for 4-HBA transfer onto monolignols and the transferase(s) involved remain to be elucidated as well [29].

Gallate is a derivate of 4-HBA that is obtained from the hydrolysis of plant gallotannins using microbial tannases. As a more sustainable alternative, gallate is produced biologically from 4-HBA in engineered microorganisms using a mutant version of the 4-HBA hydroxylase PobA from *Pseudomonas aeruginosa* [30, 31]. We previously showed that PobA could be functionally expressed in Arabidopsis plastids for production of protocatechuate from 4-HBA (Fig. 2) [32]. When expressed transiently in tobacco leaves, we also showed that PobA mutant can efficiently convert 4-HBA into gallate, which accumulates mainly as glucogallin (Lin et al., unpublished). This is of particular interest since gallate can be converted into epoxy resins used in several materials such as coatings, adhesives or laminates [33], or further esterified to produce the food additives E311, E312, and E313. Additionally, gallate can be easily decarboxylated to form pyrogallol, an important platform chemical used as a reducing agent in photography and dyeing agent in cosmetics. Finally, methyl gallate can be used as starting material for the synthesis of the drug precursor thebaine [34].

### Muconic acid

Muconic acid (MA) is a platform chemical used as a precursor for the synthesis of products such as adipic acid, terephthalic acid, and caprolactam, which are widely used in the nylon and thermoplastic polymer industries. Current processes for the manufacturing of these products rely on non-renewable petroleum-based chemicals, require a high energy input, and yield large quantities of toxic by-products [35]. As an alternative, the biological production of MA using engineered microorganisms and inexpensive carbohydrate feedstocks has received increasing attention over the past 20 years [36]. Most biological routes established in microbes consist in the production of catechol and its subsequent conversion into MA by ring-cleaving catechol 1,2-dioxygenase. These routes exploit the intrinsic shikimate pathway for the biosynthesis of catechol precursors such as protocatechuate, anthranilate, salicylic acid (SA), and 2,3-dihydroxybenzoic acid [37]. The SA route was recently implemented in Arabidopsis and resulted in the production of readily extractable muconic acid from plant biomass [38]. In these plants, SA pools were increased by co-expression of plastid-targeted bacterial feedback-resistant 3-deoxy-d-arabino-heptulosonate 7-phosphate synthase (AroG*) and SA synthase (Irp9). Conversion of SA into catechol and muconic acid was further achieved by co-expression of plastid-targeted bacterial SA hydroxylase (NahG) and catechol 1,2-dioxygenase (CatA), respectively (Fig. 2). Functional expression of CatA in plants was an important milestone towards developing crops that serve as production platforms for MA. Strategies for overproducing catechol precursors other than SA from the shikimate pathway (i.e., protocatechuate and anthranilate) have already been established, but their efficient conversion into catechol remains to be demonstrated. These other routes towards MA production could be more suitable in the case of crops that trigger stress responses upon SA signaling. Nevertheless, the SA route toward production of bio-derived muconic acid could be appropriate for bioenergy crops from the Salicaceae family (e.g., poplar, willow), which are particularly productive at synthesizing multiple SA-derived compounds.

Besides its conversion into MA, catechol obtained from engineered bioenergy crops could represent a value-added product since it is a starting material for the manufacturing of insecticides (e.g., carbofuran and propoxur), fragrances, drugs, and polymerization inhibitors [39]. Recently, catechol was converted to a deep eutectic solvent (DES) that was found to be effective for the pretreatment of plant biomass and facilitate the removal of lignin prior enzymatic saccharification [40].

### Protocatechuate

Protocatechuate (PCA) represents a valuable coproduct that has potential to add value to biomass of bioenergy crops as it possesses several pharmacological applications related to its antioxidant activities and anti-inflammatory properties [41]. In addition, several studies reported on the biological upgrade of PCA using engineered microbial strains. For these approaches, no purification step of PCA from biomass is required since engineered microbial strains utilize the various components of biomass hydrolysates for growth while funneling PCA into valuable chemicals. As examples, engineered *Pseudomonas* strains have been developed for efficient conversion of PCA into beta-ketoadipic acid, muconolactone, and 2-pyrone-4,6-dicarboxylic acid [42, 43], and engineered *Rhodosporidium* strains have been designed for conversion of aromatics into the biofuel precursor bisabolene [18].

Although PCA is commonly found in several plant species [41], no biosynthetic routes have been described so far. Two engineering approaches have resulted in higher PCA titers in plants. Expression of 3-dehydroshikimate dehydratase (QsuB) from *Corynebacterium glutamicum* allows conversion of 3-dehydroshikimate into PCA [44]. Co-expression of chorismate pyruvate-lyase (UbiC) from *E. coli* and 4-HBA hydroxylase (PobA) from *Pseudomonas aeruginosa* allows conversion of chorismate into PCA via 4-HBA (Fig. 2) [32]. For both approaches, bacterial enzymes were targeted to plastids in order to co-localize with their substrate, and PCA accumulated in green tissues mainly as conjugated forms (presumably glycosides), suggesting a transit of PCA from plastids to vacuoles and via the cytosol. In tobacco plants expressing QsuB, PCA conjugates were readily extracted from senesced biomass using aqueous methanol as solvent, and free PCA subsequently recovered after an acid hydrolysis step was efficiently upgraded biologically into muconic acid using an engineered *E. coli* strain [45]. In connection to these observations, a techno-economic analysis in sweet sorghum assessed that achieving PCA titers of 5% DW in biomass could reduce the minimum selling price of cellulosic ethanol obtained from sorghum bagasse if PCA is efficiently converted biologically into muconic acid as coproduct [46]. Interestingly, PCA was shown to be a competitive inhibitor of the lignin biosynthetic enzyme hydroxycinnamoyl-CoA:shikimate hydroxycinnamoyl transferase (HCT), therefore, increasing PCA in crops is also a strategy for reducing lignin and improving biomass digestibility [32]. So far, the QsuB approach aiming at overproducing PCA in biomass has been successfully translated to bioenergy crop such as poplar (Shawn Mansfield, University of British Columbia, personal communication), sorghum and switchgrass (Eudes et al., unpublished).

### 2-Phenylethanol

2-Phenylethanol (2-PE) is found in essential oils of several plant species such as rose, carnation, hyacinth, and jasmine. It is one of the most used chemicals for fragrances and flavors due to its pleasant rose-like aroma, and it also represents a precursor for producing the flavoring agent 2-phenylethyl acetate. 2-PE also finds applications as antibacterial and antifungal agent [47]. Other studies have explored the use of 2-PE for the production of ethyl benzene, a monocyclic aromatic hydrocarbon important in the petrochemical industry for the synthesis of styrene, which is the precursor to the common plastic material polystyrene [48]. Currently, 2-PE, styrene, and ethyl benzene are produced from petrochemical sources. There is, however, a demand for bio-based 2-PE in the case of flavor and fragrance applications, with a selling price about two orders of magnitude higher compared to synthetic 2-PE [49].

Overproduction of 2-PE has been reported in plants. Transgenic hybrid poplar co-expressing tomato phenylacetaldehyde reductase (PAR) and rose phenylacetaldehyde synthase (PAAS), both under the control of a constitutive promoter, produced more than 3.5% DW of 2-PE in leaves (Fig. 2). 2-PE accumulated as a glucoside form that was readily extractable using methyl *tert*-butyl ether as solvent [50]. Interestingly, a similar work conducted in Arabidopsis showed reduction of lignin content and improved biomass saccharification in addition to 2-PE production in engineered lines co-expressing PAR and PAAS genes [51]. Therefore, the approach may have the potential to reduce biomass recalcitrance for production of simple sugars in combination with the delivery of a valuable coproduct. The agronomic performances of such bioenergy crops remain to be evaluated. It would be interesting for example to assess their resistance to insect herbivores considering that certain poplar species produce 2-PE as defense compound [52].

### Eugenol

Eugenol, an aromatic compound found in essential oils from clove, nutmeg, cinnamon, basil and bay leaf, is used as flavoring agent in perfumes, food and cosmetics. Actually, commercial eugenol is made from the refining of these oils obtained by steam distillation [53, 54]. Eugenol is also used as a local antiseptic and analgesic for dentistry, and other pharmaceutical applications for eugenol or its derivatives have been explored. Moreover, eugenol can serve as substrate for biological conversion into vanillin through a two-step biotransformation process that uses engineered *E. coli* [55].

Eugenol is a phenylpropanoid that derives from coniferyl alcohol, which is also the precursor to guaiacyl units in lignin. In flowers and leaves that produce eugenol, coniferyl alcohol is converted into coniferyl acetate by coniferyl alcohol:acetyl-CoA transferase (e.g., petunia PhCAAT and creosote bush LtCAAT) [56, 57], which is in turn converted into eugenol by eugenol synthase (e.g., creosote bush LtAPS, sweet basil ObEGS and petunia PhEGS) (Fig. 2) [58, 59]. Constitutive expression of PhCAAT in hybrid aspen successfully resulted in the production of eugenol (> 80 μg/g fresh weight (FW)) and its glucosides, which were extracted from biomass using hexane and aqueous methanol solvents, respectively. Interestingly, co-expression of PhCAAT with PhEGS did not improve eugenol yields further [60]. Similarly, constitutive co-expression of LtCAAT and LtAPS in hybrid poplar resulted in the production of eugenol glucoside (~ 0.4% DW). Preliminary data did not reveal any significant differences for lignin content in eugenol overproducing trees, but an early flowering observed after 4‐year field trial growth suggested some developmental modifications [61].

### *p*-Coumarate

*p*-Coumarate (*p*CA) is a hydroxycinnamate that serves as precursor for manufacturing valuable chemicals. It includes for example 4-vinylphenol (or *p*-hydroxystyrene, *p*-HS), a versatile petroleum-derived platform chemical used to produce polyvinylphenol (PVP), which is employed in photoresist materials, elastomers, resins and coatings. *p*-HS is also used to produce flavoring and fragrance substances in food, beverage and perfume industries. Several microbial strains have been developed for bio-based production of *p*-HS using *p*CA as direct precursor [62, 63]. Other engineered microbial strains have been designed to upgrade *p*CA into resveratrol, an antioxidant with potential benefits for human health [64–66], as well as the biofuel precursor bisabolene [18], the platform chemical muconic acid [67], the biodegradable polyester precursor lactic acid [68], and polyhydroxyalkanoate polyesters [20, 69, 70]. Moreover, *p*CA can be transformed chemically to deep eutectic solvents potentially useful for biomass pretreatments [40] and converted to different types of high-performance polymers [71, 72].

Biomass from bioenergy crops, especially grasses such as switchgrass, corn, and sorghum, contains non-negligible amount of *p*CA which occurs on the hydroxyl group on the γ-carbon of lignin unit side chains, mostly on syringyl units [73]. Tyrosine ammonia-lyase (TAL) catalyzes the conversion of tyrosine into *p*CA, and expression of a bacterial TAL in Arabidopsis was sufficient to increase the accumulation of soluble *p*CA derivatives such as anthocyanins and flavonoids [74]. Therefore, higher content of *p*CA in cell wall biomass may be achieved by concomitantly boosting *p*CA production from tyrosine and promoting *p*CA transfer onto lignin monomers. Engineering strategies that enhance tyrosine in plants include expression of feedback‐insensitive 3‐deoxy‐D‐arabino‐heptulosonate 7‐phosphate synthase (AroG*) from *E. coli*, which resulted in a ~ 20-fold increase in tyrosine content [75], and expression of an isoform of arogenate dehydrogenase with relaxed sensitivity to tyrosine negative feedback inhibition (ADHα from *Beta vulgaris*), which led to ~ 100-fold increase in tyrosine accumulation (Fig. 2) [76]. Moreover, several enzymes named *p*-coumaroyl-CoA: monolignol transferase (PMT) and involved in the transfer of the CoA-activated form of *p*CA onto monolignols have been discovered in rice, Brachypodium, and maize (Fig. 2) [77–79]. Expression of these transferases in Arabidopsis and poplar resulted in increases of cell wall-bound *p*CA, which is otherwise found in low amounts in these two eudicots plants [80, 81]. Recently, overexpression of the maize PMT gene in maize led to a ~ 40% increase in lignin-bound *p*CA, which was released from biomass upon alkaline treatment [82]. Interestingly, higher amount of *p*CA attached to lignins in Arabidopsis resulted in increased lignin solubility under alkaline treatment of biomass [81]. These observations are relevant considering that base-catalyzed depolymerization liquors generated from biomass are rich in *p*CA and other aromatic compounds that are suitable for microbial conversion [83, 84]. Alternatively, *p*CA can be recovered and purified as coproduct from the alkaline pretreatment stream after ethanol separation and precipitation under acidic conditions [82]. For such a biorefinery concept, a techno-economic analysis indicates that *p*CA content in plant biomass should be at least 5% DW in order to be economically attractive at current *p*CA market price [82]. Other purification methods potentially involve membrane fractionation [85] or ultrafiltration coupled to affinity adsorption [86]. Finally, in switchgrass, overexpression of a transferase from rice (OsAT10) was shown to increase cell wall-bound *p*CA and to enhance biomass saccharification efficiency [87]. Although being part of the same ‘BAHD’ enzyme family that contains PMT transferases, OsAT10 belongs to a different clade and its substrates remain to be identified.

### Caffeate and ferulate

As described previously for *p*CA, caffeic and ferulic acids are valuable building blocks for the manufacturing of advanced polymers, especially polyesters, which have a wide range of applications [88]. Moreover, in the case of ferulic acid, approaches for biological conversion into important chemicals such as vanillin [89, 90], polyhydroxyalkanoate [69], and muconic acid [91, 92] have also been reported. Ferulic acid was also recently used as starting material for the synthesis of ilicifoline, a dimeric berberine alkaloid with potential pharmacological and therapeutic effects [93]. Caffeic acid and its phenethyl ester (CAPE) are used in the pharmaceutical and cosmetic industries due to their anti-oxidant, anti-aging and anti-carcinogenic activities. For these markets, caffeic acid is extracted from plants whereas its ester is produced by chemical synthesis [94].

Caffeic and ferulic acids are two hydroxycinnamates that derive from the general phenylpropanoid pathway. Caffeic acid, in addition to being an intermediate of the monolignol biosynthetic pathway for lignin, is found as a constituent of lipid polymers that form cuticle and suberin [95]. It is also part of several conjugated molecules such as chlorogenic acids, clovamide, rosmarinic acid, and CAPE, as well as polyamine conjugates. Ferulic acid is also found in lipid polymers, polyamines, and certain chlorogenic acids and anthocyanins. Hemicelluloses represent an important source of ferulate in plant biomass: ferulate is esterified to arabinose residues in xylan and participate in crosslinking between hemicellulose and lignin [73, 96]. The release of ferulate from sugarcane biomass has been described using alkaline-sulfite chemi-thermomechanical pretreatment [97].

Enhancing ferulate and caffeate contents in plant biomass can be achieved by overexpressing BAHD transferases involved in the attachement of ferulate and caffeate moieties to polymers like suberin and cutin, or to compounds like chlorogenic acids [98, 99]. However, targeting xylan to increase ferulate amount in biomass could represent a more attractive strategy considering the large amount of hemicellulose present in cell walls. In sorghum, overexpression of CCoAOMT, an O-methyltransferase that methylates caffeoyl-CoA to generate feruloyl-CoA, led to an increase of cell wall-bound ferulates in biomass [100]. Similarly, overexpression in Brachypodium of a BAHD feruloyl-CoA transferase potentially involved in arabinoxylan feruloylation resulted in higher content of ferulates in cell walls [101]. These observations suggest that increasing both the pool of feruloyl-CoA and the expression of xylan-specific feruloyl-CoA transferases could lead to higher amount of easily cleavable ferulate esters in biomass of bioenergy crops.

### Vanillin and syringaldehyde

Vanillin is extensively used as flavor and fragrance in food, beverages, cosmetics, pharmaceutical formulations, and homecare products [102]. It is also a precursor for the manufacturing of bio-based epoxy resins [103], and, along with syringaldehyde, can be used to produce polymers with high thermostability [104]. In addition, syringaldehyde has bioactive properties and is therefore used in pharmaceuticals (e.g., trimethoprim antibiotics), foods, and cosmetics [105]. Lastly, vanillin represents a suitable precursor for biological conversion into platform chemicals such as 2-pyrone-4,6-dicarboxylic acid and muconic acid [45, 106].

Despite its economic importance, the vanillin/syringaldehyde biosynthetic pathway in plants has not been fully elucidated [107]. Nevertheless, several engineering approaches have resulted in augmentations of these two aromatics in biomass. Plants affected in the lignin biosynthetic enzyme cinnamyl alcohol dehydrogenase (CAD) are known to accumulate significantly higher amounts of vanillin and syringaldehyde [108]. In CAD-deficient pine and poplar, higher quantity of these hydroxybenzaldehydes is released from biomass after alcohol- or alkaline-based extractions [109, 110]. Furthermore, the effectiveness of hydrothermal treatments at releasing vanillin and syringaldehyde from biomass of CAD mutant plants was recently evidenced [111, 112]. Development of environmentally friendly processes towards valorization of lignin streams generated in biorefineries has garnered interest, and it would be interesting to evaluate the yield of vanillin and syringaldehyde obtained from the depolymerization of lignins that derive from CAD mutants [113]. Interestingly, it has been demonstrated that the ratio of vanillin to syringaldehyde in biomass can be modulated by altering the expression of coniferaldehyde 5-hydroxylase (CAld5H) in a CAD mutant background (Fig. 2) [114]. Finally, in sorghum, overexpression of MYB transcription factor (SbMyb60) that induces monolignol biosynthesis resulted in higher amount of syringaldehyde crosslinked to cell walls [115].

### Indican

Indican (indoxyl-beta-d-glucoside) is a metabolite naturally occurring at ~ 1–2% FW in leaves of *Indigofera* plants and other species such as the Japanese indigo plant (*Persicaria tinctoria*) [116]. Upon beta-glucosidase activity, indican is hydrolyzed to indoxyl which spontaneously undergoes oxidative dimerization to form crystalline indigotine, an important blue chemical used as indigo dye [117]. Several thousand tons of synthetic indigo are produced each year from non-renewable petroleum-derived chemicals for the textile industry. Especially, indigo is chemically synthesized from aniline, a toxic aromatic derived from benzene, which involves the use of hazardous compounds such as formaldehyde, hydrogen cyanide, sodamide, and strong bases [118]. Alternatively, the water extraction process of indican from *Indigofera* plant species and its conversion into indigotine has been practiced in various forms for hundreds of years throughout the world, and current demand for natural indigo is expanding [119]. Bioenergy crops, because of their higher biomass yield and amenability to metabolic engineering, could be used as platform for bio-based and sustainable indican production. Considering that around 50 thousand tons of indigo are produced annually worldwide, 5 million tons of engineered biomass containing indoxyl at 2% DW could potentially supply the entire market each year, with the assumption of 100% extraction and recovery efficiencies. Based on the estimates from the U.S. Department of Energy’s 2016 Billion-Ton Report, ~ 27 million tons of switchgrass could be economically available annually by 2040 at an offered farmgate price ≤ 40$ per dry ton and considering a baseline scenario of 1% yield growth per year [120]. This suggests that ~ 18.5% of switchgrass grown in the U.S. in the future would need to be engineered for indoxyl production (i.e., 2% DW) to potentially supply the entire indigo current global market.

Indican synthesis was achieved in tobacco by expression of indole synthase (BX1) from maize and a cytochrome P450 monooxygenase (CYP2A6) from human (Fig. 2) [121]. The activity of endogenous glucosyltransferases allowed conversion of indoxyl into indican, which prevented formation of blue indigotine crystals *in planta*. Alternatively, as shown in transient expression studies in tobacco, enhancement of indole synthesis can be achieved by expressing a tryptophanase (TnA) from *E. coli*, which also resulted in indican biosynthesis when co-expressed with CYP2A6 (Fig. 2) [122]. Therefore, engineering strategies aiming at increasing tryptophan content could be leveraged for higher production of indican in bioenergy crops. For example, expression in Arabidopsis of feedback‐insensitive 3‐deoxy‐d‐arabino‐heptulosonate 7‐phosphate synthase (AroG*) from *E. coli* led to ~ 2.5-fold increases in tryptophan content [123]. Similarly, expression in rice of feedback‐insensitive anthranilate synthase (OASA1D) from rice resulted in 35-fold increases in tryptophan accumulation (Fig. 2) [124].

### Aminobenzoates

*p*-Aminobenzoic acid (PABA) and its derivatives are commonly used as ultraviolet-B filters in cosmetic sunscreens. PABA also has applications as cross-linking agent for polyurethane resins and dyes [125]. Its derivative ethyl PABA (anesthesin) is a local anesthetic of low toxicity used in dentistry, and potassium PABA (Potaba) has applications in pharmacy due to its anti-inflammatory and antifibrotic activities. PABA is currently produced chemically from 4-nitrobenzoic acid, which itself is produced from petroleum-derived toluene. 2-Aminobenzoic acid (anthranilic acid) is an intermediate in the production of the artificial sweetener saccharin [126] and can also act as a non-toxic corrosion inhibitor for metallic materials [127]. In addition, anthranilic acid esters are widely employed for the synthesis of azo dyes, pharmaceuticals (e.g., loop diuretics and fenamates), perfumes, and potential insect repellents [125, 128].

In plants, PABA derives from a two-step conversion of chorismate catalyzed by 4-amino-4-deoxychorismate synthase (ADCS) and lyase (ADCL), respectively (Fig. 2) [129]. Anthranilic acid derives from conversion of chorismate by anthranilate synthase which is composed of alpha and beta subunits [130, 131]. Arabidopsis plants affected in anthranilate phosphoribosyl transferase (PAT) activity accumulate large quantities of anthranilic acid and its glycosides, but also exhibit a dwarf phenotype compared to wildtype [132]. Interestingly, in order to alleviate the negative effect on biomass yield, we observed that overexpression of a feedback‐insensitive anthranilate synthase (TRP5) in an Arabidopsis background deficient in PAT activity was able to restore growth characteristics similar to wildtype plants while maintaining elevated levels of anthranilic acid and its glycosides (Berthomieu et al., unpublished) (Fig. 2). These observations, in conjunction with the opportunity of exploiting anthranilate-specific UDP-glucosyltransferases and BAHD acyltransferases to favor the formation of anthranilic acid conjugates that accumulate in vacuoles or cell walls [44, 133, 134], could guide the design of bioenergy crops delivering anthranilic acid as valuable coproduct. Finally, several studies reported on the overexpression of ADCS in fruits and seeds of various crops to greatly enhance PABA as part of folate biofortification strategies [135], and a PABA-specific glucosyltransferase that mediates vacuolar storage of PABA-glucose ester has been identified [136].

## Biochemicals derived from the isoprenoid pathways

Terpenes and terpenoids are the largest and most diverse class of specialized metabolites (> 80,000 compounds [137]), which possess versatile biological and ecological functions for regulating plant development and responses to biotic and abiotic stresses. In general, the structural diversity of terpenes depends on the number of isoprene (C_5_H_8_) moieties that constitute them. With the exception of steroids (C_27_), terpenes can be subdivided into hemi- (C_5_), mono- (C_10_), sesqui- (C_15_), di- (C_20_), sester- (C_25_), tri- (C_30_), tetra- (C_40_) and polyterpenes (C_5n_, *n* > 8), based on the number of isoprene units [138]. In contrast to terpenes, which comprise solely hydrocarbons, terpenoids are more structurally diverse and consist of oxygen-containing or chemically modified terpene analogs [139]. For convenience, all the terpenes and terpenoids described in this review will be named “isoprenoids”. In this section, we will review several isoprenoids considered as potential bioproducts with commercial value for the production of nutraceuticals, flavors/scents/colors, biofuels and biopolymers.

### Isoprenoids biosynthetic pathways

**Fig. 3:**
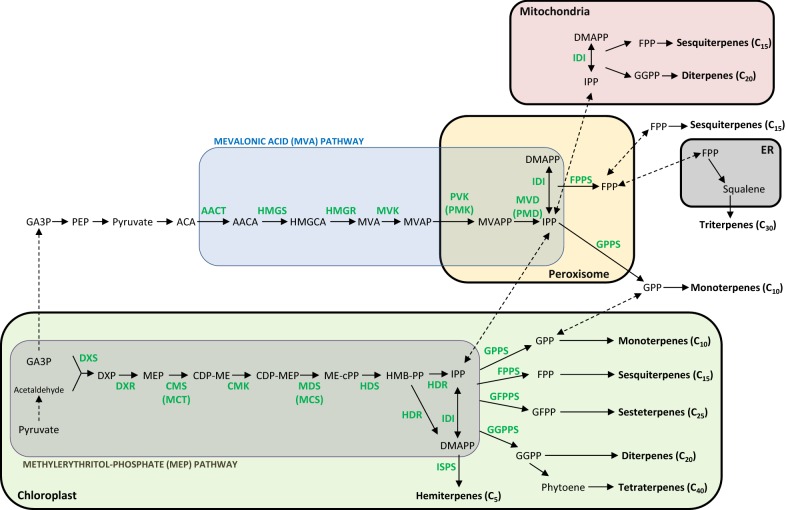
A depict of isoprenoid biosynthesis from cytosolic MVA and plastidial MEP pathways. AACA, acetoacetyl-CoA; AACT, acetoacetyl-CoA thiolase; ACA, acetyl-CoA; CDP-ME, 4-diphosphocytidyl-2-C-methylerythritol; CDP-MEP, CDP ME 2-phosphate; CMK, CDP-ME kinase; CMS/MCT, CDP-ME synthase; DMAPP, dimethylallyl diphosphate; DXP, 1-deoxy-d-xylulose 5-phosphate; DXR, DXP reductoisomerase; DXS, DXP synthase; ER, endoplasmic reticulum; FPP, farnesyl diphosphate; FPPS, farnesyl diphosphate synthase; GA3P, glyceraldehydes-3-phosphate; GFPP, geranylfarnesyl diphosphate; GFPPS, geranylfarnesyl diphosphate synthase; GGPP, geranylgeranyl diphosphate; GGPPS, geranylgeranyl diphosphate synthase; GPP, geranyl diphosphate; GPPS, geranyl diphosphate synthase; HDR, HMBPP reductase; HDS, HMB-PP synthase; HMB-PP, 1-hydroxy-2-methyl-2-(E)-butenyl 4-diphosphate; HMGR, HMG-CoA reductase; HMGCA, 3-hydroxy-3-methylglutaryl-CoA; HMGS, HMG-CoA synthase; IDI/IPI, IPP isomerase; IPP, isopentenyl pyrophosphate; ISPS, isoprene synthase; MCS/MDS, ME-cPP synthase; ME-cPP, ME 2,4-cyclodiphosphate; MVA, mevalonate; MVAP, mevalonate-5-phosphate; MVAPP, mevalonate 5-diphosphate; MVD/PMD, MVAPP decarboxylase; MVK, MVA kinase; PEP, phosphoenolpyruvate; PVK/PMK, MVAP kinase; MEP, 2-C-methyl-d-erythritol-4-phosphate

Isoprenoids are all derived biosynthetically from the five-carbon precursors isopentenyl pyrophosphate (IPP) [140, 141] and its isomer dimethylallyl diphosphate (DMAPP) [142], for which the ratio is controlled by IPP isomerase (IDI/IPI) [143, 144]. The biosynthesis of IPP or DMAPP in plants occurs via two spatially distinct pathways, which are the cytosolic mevalonate (MVA) pathway [145] and the plastidial methylerythritol-phosphate (MEP) pathway [146] (Fig. 3). More generally, the MVA pathway is germane to Archaea and present in most eukaryotes, fungi (*Saccharomyces cerevisiae*), plant cytoplasm, and some bacteria (enterococci, staphylococci, and streptococci); while the MEP pathway is germane to Bacteria and exists in most cyanobacteria, algae, plant chloroplasts, and some protozoa [147–149]. Studies revealed that formation of diverse terpenes, including triterpenes (steroids), brassinosteroids, ubiquinones and most sesquiterpenes originates from the MVA pathway, whereas isoprene, hemiterpenes, monoterpenoids, some sesquiterpenes, diterpenoids, tetraterpenoids, abscisic acid, strigolactones, gibberellins, prenylquinones, and the phytol tail of chlorophylls are derived from the MEP pathway [140, 150, 151]. In addition, it has been suggested that both pathways are antagonistically regulated by the circadian clock, which may be exploited as an engineering strategy to orchestrate gene expression of the two pathways for production of desired products [140]. For monoterpene biosynthesis, geranyl diphosphate (GPP, C_10_) is formed from the condensation of DMAPP with IPP, which is catalyzed by geranyl diphosphate synthase (GPPS). For sesquiterpene biosynthesis, GPP is converted with one equivalent of IPP by farnesyl diphosphate synthase (FPPS) to farnesyl diphosphate (FPP, C_15_). Subsequently, FPP further condenses with one IPP to form geranylgeranyl diphosphate (GGPP, C_20_) by GGPP synthase (GGPPS) for diterpene biosynthesis. For sesterterpene synthesis, GGPP undergoes one more condensation with IPP to produce geranylfarnesyl diphosphate (GFPP, C_25_), which is catalyzed by GFPP synthase (GFPPS). Although these common precursors (e.g., IPP, GPP, FPP, and GGPP) are synthesized by the two distinct pathways, they can be transported freely to different cellular compartments. For higher carbon skeletons of terpenes such as triterpenes and tetraterpenes, two molecules of FPP or GGPP are typically dimerized to form the precursors squalene (C_30_) and phytoene (C_40_), respectively [152] (Fig. 3). Finally, to synthesize diverse functional terpene molecules, these precursors will further undergo a variety of reactions such as isomerization, cyclization, reduction, oxidation, and conjugation.

### Engineering approaches for increasing the metabolic flux through isoprenoid biosynthesis

The amount of specialized isoprenoids in plants is low, and it is impractical to rely solely on natural plant resources for production and large-scale downstream applications [153]. In general, the structure of these compounds is too complex to achieve their chemical synthesis with high regio- and stereos- specificity [154]. Therefore, in order to produce sustainably bioactive isoprenoids at higher titers, boosting the common precursors within the MVA or MEP pathways via plant metabolic engineering represents an attractive option. The biosynthetic pathways of isoprenoids are known to be highly regulated, and enzymes that control metabolic fluxes were identified and studied towards maximizing product yields within the MVA or MEP pathways [155, 156]. For terpenoid biosynthesis, IDI/IPI and several other enzymes in the MVA pathway (HMGR, HMGS, MVK, and PMK/PVK) and the MEP pathway (DXS, DXR, and HDR) are rate-limiting enzymes [157, 158] (Fig. 3).

HMGR (EC 1.1.1.34) activity and transcript abundance can be regulated by multiple factors such as hormones, environmental signals, and metabolic needs [159, 160]. In contrast to a single HMGR gene found in animals, archaea, and eubacteria, higher plants possess multiple HMGR isozymes, which are spatially and temporally regulated at the gene expression level [161, 162]. These are 60–65 kDa in size and their structure can be divided into three regions: an N-terminal domain for subcellular compartmentalization, a central domain with two transmembrane spanning regions that can modulate protein level, and a C-terminal domain for catalytic activity [151, 163]. It has been shown that overexpression of a HMGR truncated N-terminal domain (t-HMGR) can improve the production of amorphadiene and taxadiene in *S. cerevisiae* [164, 165]. Recently, in tobacco, overexpression of biotin carboxyl carrier protein (BCCP) linked to t-HMGR by a cleavable peptide 2A increased 20–40-fold C_15_ sesquiterpenes and sixfold C_30_ β-amyrin (a triterpene), which demonstrated an new strategy to improve terpenoid production by incorporating metabolic cross-talks [166]. In addition, heterologous expression in Arabidopsis of HMGR from ginseng enhanced the production of phytosterols and the two triterpenes α-amyrin and β-amyrin [167].

HMGS (EC 2.3.3.10) is also considered to be a rate-limiting enzyme [168] whose activity is inhibited by both substrate (acetoacetyl-CoA) and products (HMG-CoA and HS-CoA) [169]. Several functional HMGS have been cloned or characterized from plants such as Arabidopsis [170], pine [171], rubber tree [172], ginkgo [173], maize [174], and mustard [175]. Overexpression of GlHMGS from lingzhi mushroom can boost the production of ganoderic acid in *Ganoderma lucidum* [176]. Several mutants of BjHMGS1 from brown mustard *(Brassica juncea*) have been generated [169]: one of them (H188N) is insensitive to substrate inhibition but showed an eightfold decrease in enzyme activity, whereas overexpression of BjHMGS1 (S359A) improved the production of terpenoids such as sterol [177], α-tocopherol, carotenoid, squalene, and phytosterols [178].

DXS (EC 2.2.1.7) is the first entry point of the MEP pathway and a well-known rate-limiting enzyme in bacteria and plants [157, 179, 180]. In plants, based on sequence similarity, three different classes of DXS enzymes have been proposed: class 1 contains essential enzymes to synthesize terpenoids for photosynthesis, class 2 DXS is correlated with secondary terpenoids biosynthesis, and class 3 DXS are involved in the synthesis of isoprenoids (phytohormones) [181, 182]. Manipulating the activity of DXS has been considered as an effective strategy to fine-tune the biosynthesis of terpenoids (i.e., chlorophyll, carotenoids, tocopherols, abscisic acid, or gibberellin), as demonstrated in several plants, such as Arabidopsis [183–185], tomato [186, 187], ginkgo [188], potato [189] and carrot [190]. Moreover, DXS activity is subjected to negative feedback-regulation by IPP and DMAPP, which can bind and allosterically inactivate DXS [191, 192].

Downstream of DXS in the MEP pathway, DXR (EC 1.1.1.267) also represents a potential point of regulation [193]. Several transgenic approaches evidenced positive correlations between DXR expression levels and the content of isoprenoids. For example, overexpression of DXR increased the production of plastid isoprenoids (i.e., chlorophylls, carotenoids, and taxadiene) in Arabidopsis [194] and the level of monoterpenes in leucoplasts of peppermint [195]. Similarly, overexpression of *Synechosystis* DXR in tobacco chloroplasts increased the production of isoprenoids (i.e., chlorophyll a, β-carotene, lutein, antheraxanthin, solanesol and β-sitosterol) [196]. Co-expression of DXR and solanesyl diphosphate synthase (SDS) in potato can significantly enhance the levels of solanesol compared to DXR alone [197]. In addition, higher DXR levels have been associated with a role in arbuscule development in the roots of several monocot plants (e.g., wheat, maize, rice, and barley) along with increased apocarotenoid biosynthesis [198, 199].

## Isoprenoid-derived biofuels and bioproducts

With recent advancements in plant synthetic biology, implementation in crops of multigene isoprenoid metabolic pathways to develop biofuels and bioproducts at industrial scale is becoming attainable [200–202]. The wondrous diversity and structural richness of isoprenoid molecules from plants provide a plethora of potent and useful target compounds, especially for advanced biofuels and value-added bioproducts [152, 203–206]. In relation to bioenergy crops, several efforts have been made to elucidate the genetic components that influence terpene yields, as well as to develop biomass pretreatment methods for simultaneous extraction of both terpenes and fermentable sugars [207, 208]. More generally, several methods have been developed for the extraction and isolation of terpenoids from plants [209], but these processes often remain major bottlenecks for the production of chemicals from plant biomass at industrial scale.

### Isoprenoid-derived nutraceuticals carnosic acid (C_20_H_28_O_4_, diterpenoid)

**Fig. 4:**
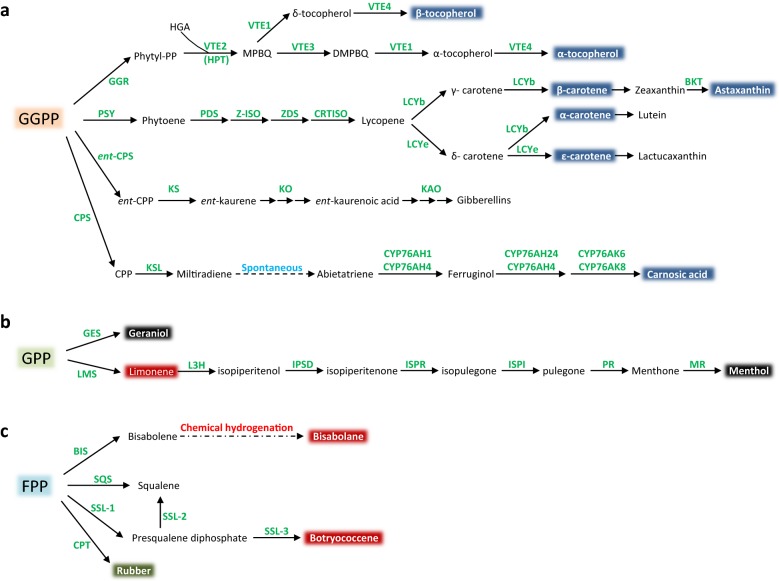
Proposed metabolic steps for the synthesis of isoprenoid-derived nutraceuticals (blue), flavors/fragrances/cosmetics (black), biofuel (red) and polymer (green). **a** Biosynthesis of selected isoprenoids from GPP. **b** Biosynthesis of selected isoprenoids from FPP. **c** Biosynthesis of selected isoprenoids from GGPP. GES, geraniol synthase; LMS, limonene synthase; L3H, limonene 3-hydroxylase; ISPD, isopiperitenol dehydrogenase; ISPR, isopiperitenone reductase; ISPI, isopulegone isomerase; PR, pulegone reductase; MR, menthone reductase (MR). BIS, bisabolene synthases; SQS, squalene synthase; SSL, squalene synthase-like enzyme; CPT, *cis*-prenyltransferase; GGR, geranylgeranyl reductase; VTE2, homogentisate phytyl transferase (HPT); VTE1, tocopherol cyclase (TC); VTE4, γ-tocopherol methyl transferase (γ-TMT); VTE3, 2-methyl-6-phytyl-1,4-benzoquinol methyl transferase; PSY, phytoene synthase; PDS, phytoene desaturase; Z-ISO, ζ-carotene isomerase; ZDS, ζ-carotene desaturase; CRTISO, carotenoid isomerase; LCTb, lycopene β-cyclase (LCYb); BKT, β-carotene ketolase; LCYe, lycopene ε-cyclase; ent-CPS, *ent*-copalyl diphosphate synthase; KS, kaurene synthase; KAO, *ent*-kaurenoic acid oxidase; KO, *ent*-kaurene oxidase; CPS, copalyl diphosphate synthase; KSL, kaurene synthase-like enzyme; HGA, homogentisate; DMBQ, 2-methyl-6-phytylbenzoquinol; DMPBQ, 2,3-dimethyl-6-phytyl-1,4-benzoquinol

Carnosic acid is considered as a nutraceutical due to its antioxidant, preservative and antimicrobial capacities, and represents an important constituent in food, beverages, cosmetic, and medicinal products [210]. It is exclusively identified in plant species of the Lamiaceae family, such as *Salvia officinalis* and rosemary *(Rosmarinus officinalis*), where its content can reach 10% DW in certain cultivars [210]. Carnosic acid and its oxidized derivative, carnosol (C_20_H_26_O_4_), are the two main bioactive components responsible for 90% of antioxidant properties of rosemary extracts [210]. Recently, their isolation from rosemary in high yield and purity in one step was developed using centrifugal partition chromatography [211]. The biosynthesis of carnosic acid is initiated by the conversion of GGPP into miltiradiene mediated by copalyl diphosphate synthase (CPS) and a kaurene synthase-like (KSL) protein. Following spontaneous oxidation of miltiradiene to abietatriene, members of the CYP76AH sub-family (CYP76AH1 and CYP76AH4) were proposed to catalyze abietatriene to ferruginol [212]. Finally, consecutive oxidations of ferruginol by CYP76AH24 or CYP76AH4 and CYP76AK6 or CYP76AK8 produce carnosic acid [213] (Fig. 4a).

### Carotenoids (tetraterpenoids)

Carotenoids are universal red, orange, and yellow pigments found in plants, algae, fungi, and photosynthetic bacteria [214]. Due to their antioxidant and cytoprotective properties, carotenoids and their derivatives (i.e., apocarotenoids) are high-value supplements for the food, feed, beverage, and nutraceuticals/nutricosmetics industries [215–217]. The global market value for carotenoids is projected to reach US $1.53 billion by 2021 with a compound annual growth rate (CAGR) of 3.9% [214, 218]. Among different types of carotenoids, astaxanthin is more bioactive than zeaxanthin, lutein, and β-carotene, which is mainly due to the presence of a keto- and a hydroxyl group on each end of its molecule [219]. Engineered Arabidopsis overexpressing an algal β-carotene ketolase (CrBKT) accumulates high amounts of astaxanthin (2 mg/g DW) in the leaves [220] and shows enhanced oxidative stress tolerance and bacterial pathogen resistance [221].

The biosynthetic pathway of (apo)carotenoids and their industrial potential have been extensively reviewed [214, 222] (Fig. 4a). In brief, the first committed step for the biosynthesis of carotenoids begins with the production of phytoene catalyzed by phytoene synthase (PSY). Phytoene is then converted to cis-lycopene through two consecutive desaturation steps, and one isomerization step catalyzed by phytoene desaturase (PDS), ζ-carotene isomerase (Z-ISO), and ζ-carotene desaturase (ZDS). Cs-lycopene is further isomerized to all-trans lycopene by carotenoid isomerase (CRTISO) [223]. Cyclization of all-trans lycopene by lycopene β-cyclase (LCYb) or lycopene ε-cyclase (LCYe) [224] creates a branch point for the synthesis of unique carotene backbones such as α-, β-, γ-, δ-, or ε-carotene. Finally, carotene molecules undergo a variety of decorations catalyzed by specific CYP450, epoxidase, ketolase or hydroxylase to produce the bioactive carotenoids [222]. Successful engineering of rice grain resulted in the accumulation of β-carotene, canthaxanthin, capsanthin and astaxanthin in the case of Golden Rice and aSTARice [225, 226]. Engineered maize grain with a high content of astaxanthin has been evaluated as fish feed supplement for effective pigmentation of rainbow trout flesh [227].

### Tocopherols (C_29_H_50_O_2_, diterpenoids)

Tocopherols, also known as vitamin E, can be found in plants, algae, photosynthetic organisms, and, even, non-photosynthetic parasites [228, 229]. In nature, there are eight forms of vitamin E: four types of tocopherols (α-, β-, γ-, δ-forms) and four types of tocotrienols (α-, β-, γ-, δ-forms), named according to the number and the position of methyl groups on the chromanol ring [230]. Among the eight forms, α-tocopherol is the most biologically active [231], while γ-tocopherol is the major form in many plant seeds and in the US diet [232]. Along with carotenoids, α-tocopherol is considered as an effective antioxidant for protecting lipids against photooxidation by scavenging reactive oxygen species. Therefore, α-tocopherol has also been considered as a nutraceutical to reduce the risk of human diseases, such as cancer, aging, and cardiovascular diseases [233, 234]. The biosynthesis of vitamin E has been systematically reviewed [231, 235]. In brief, it requires two precursors from distinct biosynthetic pathways, which are polyprenyl precursors from the MEP pathway and homogentisate (HGA) from the shikimate pathway (Figs. 2, 4a). Phytyl diphosphate (phytyl-PP) generated by geranylgeranyl reductase (GGR) [236, 237] is condensed with HGA by HGA phytyltransferase (HPT) to generate tocopherol precursors, while tocotrienol precursors result from the coupling of GGPP to HGA mediated by HGA geranylgeranyltransferase (HGGT) [230, 238]. These precursors undergo cyclization by tocopherol cyclase (TC) and methylation by methyltransferase (MT) to produce the δ- and γ-form, respectively. Additional methylations catalyzed by γ-TMT generate the α- and β-forms of tocopherols. In Arabidopsis, VTE2, VTE3, VTE1 and VTE4 are the enzymes corresponding to HPT, MT, TC, and γ-TMT [238]. Exogenous expression of HvHGGT from barley in Arabidopsis resulted in 10- to 15-fold increase in total vitamin E antioxidants (i.e., tocotrienols plus tocopherols), while in corn seeds, sixfold increase was achieved using the same strategy [239]. Moreover, in sorghum, HvHGGT overexpression stacked with carotenoid biosynthesis can further enhance the stability of provitamin A by mitigating β-carotene oxidative degradation [240]. Similarly, a brown seed mutant caused by the deletion of homogentisate dioxygenase (HGO) shows reduced HGA catabolism and enhanced production of vitamin E [241]. As for the engineering cases previously cited for carotenoids, enhancement of tocopherol synthesis in non-reproductive green tissues of bioenergy crops remains to be demonstrated.

### Isoprenoid-derived flavors and fragrances

The global market value of flavors and fragrances will reach US $36.6 billion by 2024 with a CAGR of 4.3% (2019–2024) [242]. For industrial products, flavors and fragrances consist of essential oils (EO) in which isoprenoids are the principal components responsible for their characteristic scents [243–245], including monoterpenoids and sesquiterpenoids such as linalool, valencene, nootkatone, and santalol [204, 246]. Because of the extensive applications of EO in fragrance, but also as insect repellents and antimicrobial agents, their global market value is expected to reach US $11.67 billion by 2022 [247].

### Geraniol (C_10_H_18_O, monoterpenoid)

Geraniol is the major components of EO of several plants, such as geranium, lemongrass rose, palmarosa, and citronella [248]. It has been used as an additive in the food and beverage industries because of its pleasant rose-like flavor, but also as natural pest control and antimicrobial agent due to its repellent and microbiocidal properties [249]. Geraniol is one of the key ingredients for the global flavor and fragrance products, and its market was valued at US $18.6 billion in 2015 [248]. However, the content of geraniol is in negligible concentrations in most plants, which provide an opportunity for its overproduction using metabolic engineering [250].

Geraniol biosynthesis occurs via the MEP pathway from GPP and can be enhanced by overexpression of geraniol synthase (GES) when the supply of GPP is sufficient [251–254] (Fig. 4b). A large-scale statistical experimental design was performed to determine the essential cultivation parameters for geraniol production using tobacco cell suspension cultures. Under optimized conditions, cells harboring the GES from *Valeriana officinalis* (VoGES) was reported to produce geraniol at ~ 5.2 mg/L after 12 days of cultivation [255]. Overexpression of VoGES in hairy roots of tobacco led to the production of geraniol and its derivatives in comparable titers, ranging from ~ 150 μg/g DW [256] to ~ 200 μg/g DW [257]. In tomato fruits, co-expression of a noncatalytic small subunit of GPPS (GPPS-SSU) from snapdragon and ObGES from basil (*Ocimum basilicum*) enhanced the production of geraniol 6.9- and 19.2-fold compared to the ObGES and GPPS-SSU parental lines, respectively [258]. Similarly, in *Catharanthus roseus*, accumulation of secologanin, a geraniol derived-monoterpene indole alkaloid, was achieved upon overexpression of CrGES along with a bifunctional CrG(G)PPS [259, 260]. In addition, a combinatorial production of geraniol in tobacco has been examined using different subcellular compartments (i.e., plastids, cytosol or mitochondria): results showed that plastid-targeted VoGES combined with expression GPPS from *Picea abies* (PaGDPS1) for GPP overproduction lead to the highest production of geraniol [261].

Furthermore, considering that geraniol is a volatile organic compound, several promising approaches for its sequestration have been reported. A UDP-glucosyltransferases from kiwifruit (*Actinidia deliciosa*), AdGT4, showed significant activity towards geraniol, with the capacity to glycosylate geraniol when transiently expressed in tobacco leaves, and to enhance the production of geraniol glycosides up to ~ 0.5 μg/g FW when expressed in tomato fruits [262]. Recently, a promising biotransformation mechanism of geraniol to methyl geranate via geraniol glucoside has been demonstrated in *Achyranthes bidentata* upon methyl jasmonate elicitation [263].

### Menthol (C_10_H_20_O, monoterpenoid)

Menthol is a major constituent of EO in peppermint (*Mentha piperita*, 30–55%) and cornmint (*Mentha arvensis,* 70–90%), the latter representing a natural source for the production of menthol crystals and natural menthol flakes by simple freeze-crystallization [264, 265]. Menthol is used in flavor and fragrance products for its cooling and refreshing sensation. It has an increasing global demand of 30,000 MT/year [153] and a market value estimated at US $3.85 billion in 2018, which is expected to reach US $5.59 billion by the end of 2025 with a CAGR of 4.8% (2019–2025) [266, 267].

The biosynthesis of menthol is proposed to be catalyzed by eight enzymes [268] (Fig. 4b): firstly, the conversion of GPP from GPPS to 4*S*-limonene is catalyzed by limonene synthase (LMS). Second, 4*S*-limonene is hydrolyzed to *trans*-isopiperitenol by limonene 3-hydroxylase (L3H, CYP71D13/15). Third, *trans*-isopiperitenol is dehydrogenated to isopiperitenone by *trans*-isopiperitenol dehydrogenase (ISPD). Subsequently, isopiperitenone undergoes three steps of reduction and one step of isomerization to produce menthol, which are catalyzed by isopiperitenone reductase (ISPR) [269], isopulegone isomerase (ISPI), pulegone reductase (PR) [269] and menthone reductase (MR) [270]. Several genetic engineering approaches with these genes in peppermint led to improved oil compositions and menthol yield [271]. Recently, an engineered bacterial ketosteroid isomerase (KSI) with ISPI activity has been generated and could be useful for heterologous production of menthol considering that plant ISPI remains unidentified [272].

### Isoprenoid-derived biofuel precursors

Several isoprenoids represent potential biofuel precursors because of their high energy density due to their cyclic nature, high octane/cetane numbers, greater molecular stability under high pressure related to their methyl branching, low freezing point through reduced molecule stacking, and high heat of combustion [205, 273]. Certain isoprenoids and their hydrogenated derivatives have been evaluated and proposed as biofuel precursors or fuel-alternatives, including monoterpenes (i.e., pinene dimers, camphene, limonene, myrcene and ocimene, and linalool), sesquiterpenoids (i.e., farnesane and bisabolene), and triterpenes (i.e., botryococcene) [274, 275].

### 4*R*-Limonene (C_10_H_16_, monoterpene)

4*R*-Limonene, a colorless cyclic monoterpene, is the major constituent (30–95%) of the *Citrus* essential oil contained in the fruit’s outer peel [276]. Several methods have been developed to extract efficiently *Citrus* essential oil, such as hydro-distillation, cold-press, instant controlled pressure drop, and steam explosion [277]. The enantiomer 4*S*-limonene is abundant in peppermint, spearmint, and perilla [278], and its biosynthesis has been extensively studied [279]. Because of a fairly high boiling point (176 °C) and high standard enthalpy of combustion (− 6100 kJ/mol), limonene represents a promising high-density isoprenoid-derived biofuel [267]. The biosynthesis of limonene requires two biosynthetic enzymes for the formation of GPP by GPPS and cyclization of GPP into limonene catalyzed by LMS [280]. Several attempts have been made to engineer limonene production in plants via overexpression of 4*S*-LMS (Fig. 4b). In peppermint, overexpression of spearmint 4*S*-LMS increased the content of menthone/menthofuran/pulegone and reduced menthol content [281], whereas its overexpression in spike lavender produced high limonene amount in the youngest leaves and revealed a possible developmental regulation of essential oil composition among the transgenic plants [282]. Overexpression of LMS from *Perilla frutescens* (PfLMS) in tobacco leaves led to higher limonene production in the case of plastid-targeted PfLMS (143 ng/g FW) compared to cytosol-targeted PfLMS (40 ng/g FW) [283]. Transgenic eucalyptus (*Eucalyptus camaldulensis*) constitutively expressing plastidic or cytosolic PfLMS produced 2.6- and 4.5-times more limonene in leaves, respectively [284]. Overexpression and plastid targeting of both Arabidopsis GPPS and *Citrus* LMS in tobacco increased limonene content 10–30 fold compared to the cytosolic-targeting strategy [285]. Recently, the overproduction of limonene has been successfully demonstrated in oilseed crop (*Camelina sativa*) by overexpressing LMS under the control of Arabidopsis promoters [286]. A recent study indicated that reaching limonene titers of 2.2% DW in bioenergy crops and extracting this coproduct from biomass at 70% efficiency could positively impact the economics of advanced biofuels [8].

### Bisabolene (C_15_H_24_, sesquiterpene)

Bisabolene has three isoforms (α-, β- and γ-bisabolene), which can be found in EO of plants including *Matricaria chamomilla, Duguetia gardneriana,* opopanax, and ginger [287, 288]. Recently, the chemically hydrogenated product of bisabolene (i.e., bisabolane) has been proposed as a promising diesel D2 replacement because of its cetane carbon number similar to that of diesel fuels, better cold property, and high energy density [289].

As a type of sesquiterpene, the biosynthesis of bisabolene requires two condensations catalyzed by GPPS and FPPS to form FPP, while FPP is subsequently converted to bisabolene by bisabolene synthase (Fig. 4c). Five plant bisabolene synthases were screened for bisabolene production and α-bisabolene synthase from *Abies grandis* (Ag1) was functionally expressed in *E. coli* and *S. cerevisiae* [289]. In plants, overexpression of tomato α-zingiberene synthase (ZIS) elevated the level of bisabolene (4–148 ng/g FW) [290].

### Botryococcene (C_34_H_58_, triterpene)

*Botryococcus braunii* is a freshwater, colonial green microalgae that represents a potential species for the production of biofuel and bioproduct precursors due to its accumulation of triterpene oil, especially squalene, botryococcene and their methylated forms [291]. The accumulated hydrocarbon oils can represent up to 86% DW and are stored in intracellular oil bodies and the extracellular matrix [292].

Botryococcene is a valuable precursor for producing chemicals and high quality fuels (gasoline and jet fuel) by standard hydrocracking and distillation at high yields (97%) [293]. Biosynthesis of botryococcene in *B. braunii* has been elucidated [294]. Unlike squalene synthesis which involves the condensation of two FPP molecules at their carbon 1, botryococcene biosynthesis is achieved by coupling two FPP molecules at their carbon 1 and 3 mediated by squalene synthase-like enzyme (SSL-1) to form presqualene diphosphate (PSPP), which is then converted to C_30_ botryococcene by SSL-3 (Fig. 4c). C_30_ botryococcene is further methylated to produce C_31_, C_32_, C_33_, C_34_, C_36_ and C_37_ botryococcenes [295]. Two successful examples for biosynthesis and accumulation of botryococcene were achieved using plants as green factories. In transgenic tobacco, up to 544 μg/g FW of botryococcene was produced when biosynthesis was directed to chloroplasts, and methylated bioproducts were obtained by introducing triterpene methyltransferases from *B. braunii* [291]. Genetic engineering in *Brachypodium* resulted in higher titers of botryococcene (> 1 mg/g FW) and healthier plants were obtained using the cytosolic-targeting strategy instead of a plastid-targeting approach [296]. Although these plant engineering efforts are promising, improving growth rates and certain physiological characteristics of *B*. *braunii* strains represent concrete research avenues towards converting this natural host into a biorefinery organism for industrial production of long-chain non-oxygenated hydrocarbons [297].

### An isoprenoid-derived polymer precursor: natural rubber ((C5H8)_n_)

Isoprenoid-derived precursors represent valuable building blocks for the production of polymers and industrial materials [298, 299]. Due to the complexity of plant biomass, it is difficult to isolate pure macromolecules; however, biosynthesis and purification of bio-based monomers for functionalization and copolymerization are simpler and cheaper. Unlike ring-opening polymerization processes, terpenes can be polymerized using radical initiators [300].

Natural rubber (NR), with an expected annual global consumption of 16.5 megatons by 2023 [301], is an industrially important isoprenoid-derived polymer because of its superior thermomechanical properties in elasticity, resilience, heat and cold resistance compared to other synthetic polymers [302]. Milky latex made by specialized laticifer cells in the bark phloem of perennial rubber tree (*Hevea brasiliensis*) is the main commercial source for NR (*cis*-1,4 polyisoprene) [303]. In general, most rubber products are made from two major types of raw materials from *Hevea* latex, which are liquid latex concentrate (60% v/v of polyisoprene) and solid dry rubber. Versatile commercial commodities such as gloves, balloons and catheters are made from liquid latex concentrate, while tires, tubing, hoses, footwear are from solid dry rubber [303].

The biosynthesis of rubber can be simply divided into two modules: a first module involves the biosynthesis of isoprenoid precursors (IPP and DMAPP), which has been discussed previously. In *H. brasiliensis*, although the existence of HbDXS and HbDXR indicates some indirect roles of the MEP pathway for rubber biosynthesis, the biosynthesis of NR is generally believed to be dependent on the MVA pathway [304–306]. A second module consists in the polymerization of oligomeric allylic diphosphates (GPP, FPP, GGPP) to *cis*-1,4 polyisoprene by rubber transferase (RT-ase or *cis*-prenyltransferase, CPT) [307] (Fig. 4c). The polymerization process of the rubber chain is initiated primarily from FPP and a CPT complex, consisting of a rubber elongation factor (REF), a small rubber particle protein (SRPP), and CPT-binding proteins (CPTBP). Finally, the termination has been proposed to be controlled by ubiquitin‐proteasome proteolysis [308, 309].

Sequencing of the rubber tree genome revealed 84 rubber biosynthesis-related genes from 20 gene families, including 18 for the MVA pathway, 22 for the MEP pathway, 15 for the biosynthesis of cytosolic isoprenoid precursors, and 29 for REF genes [310]. A review recently illustrated research developments for NR biosynthesis, advances in plant metabolic engineering towards improving NR content, and provided future perspectives for commercial development using alternative rubber crops, such as *Parthenium argentatum* (guayule) and *Taraxacum kok*-*saghyz* (rubber dandelion) [311]. Several attempts have been made to improve NR yield using transgenic plants. For example, overproduction of FPPS, GGPPS, or hexa-heptaprenyl pyrophosphate synthase (H-HPPS, a mutated form of GGPPS) in guayule showed increases of both rubber molecules and resin content in field-grown engineered guayule [312]. However, a lower molecular weight of these rubber molecules in transgenic plants was observed, indicating an endogenous metabolic flux that may lead to an insufficient IPP pool to support rubber elongation [311]. Moreover, overexpression of HbHMGR1 from the rubber tree in Arabidopsis resulted in a 50% enlargement in leaf size and more vigorous growth compared to non-transgenic plants [313]. Overexpression of HbHMGR1 increased levels of photosynthetic pigments, protein content, and, most importantly, significantly enhanced latex yield [314]. In addition, CRISPR/Cas9 genome editing has been demonstrated in rubber dandelion, which provides an avenue to accelerate the investigation of rubber biosynthesis [315]. Based on a techno-economic analysis, the accumulation of latex in bioenergy crops at titers of 2.2% DW could significantly improve the economics of second-generation biofuels [8].

## Conclusions and future perspectives

Plant metabolic engineering towards the production of chemicals has emerged as a promising approach to enhance crop value. With the advancement of biotechnological tools in both synthetic biology and plant transformation techniques, the understanding and implementation of metabolic pathways in plants became feasible [316, 317]. These technical improvements dramatically accelerate the Design–Build-Test–Learn (DBTL) cycles of plant metabolic engineering, and important increases of target biochemicals in crops are now achieved at a faster pace. One of the challenges is the testing of engineered crops under field conditions to asses stress resilience and possible yield penalty. As exemplified by the case study of crops engineered for production polyhydroxyalkanoates, subcellular compartmentalization represents an effective strategy to alleviate toxicity associated with high titers of bioproducts [318]. Similarly, promising strategies have been developed for introducing organelles that allow bioproduct sequestration and accumulation in engineered plant tissues [319]. In several cases, extraction and purification of biochemicals from plant biomass represent other important challenges to overcome for rendering biorefineries economically attractive [8]. In addition to the work conducted by plant metabolic engineers to increase titers of specific chemicals in crops, which in some instances has resulted in remarkable increases by more than two orders of magnitude after several years of research [320], an emphasis should also be given to the development of isolation and purification processes to render plant-derived chemicals economically competitive compared to their petroleum-derived equivalents. Moreover, engineering approaches and biomass processing should be evaluated with life cycle analyses to assess the environmental impacts of a specific bioproduct and inform on the types of crops to improve. Next generation of holistic biorefineries that include upstream biomass extraction step(s) prior to hydrolysis and conversion of lignocellulose are expected to beneficiate from these value-added coproduct traits implemented in bioenergy crops. The development of solvents and extraction methods compatible with existing biorefineries should enable the integration of novel streams that generate valuable coproducts while reducing recovery costs [321, 322]. Furthermore, in a concept of one-pot biomass conversion process, the release from engineered lignocellulosic feedstocks of either target bioproducts or their immediate metabolic precursors during biomass pretreatment and saccharification offers a potential for increasing final bioproduct yields, but this approach will necessitate the development of microbial strains that are tolerant to inhibitors found in lignocellulosic hydrolysates [323, 324]. For our future bioenergy crops, exploiting diverse metabolic pathways inherent to plants such as the shikimate and isoprenoid pathways will certainly contribute to the supply of several valuable biochemicals that find multiple industrial applications. Such endeavor is intended to reduce the production of fossil fuel-derived chemicals and our dependence on petroleum.

## Data Availability

Not applicable.

## Abbreviations

2-PE: 2-Phenylethanol
4-HBA: 4-Hydroxybenzoic acid
BAHD: Benzyl alcohol *O*-acetyltransferase/anthocyanin *O*-hydroxycinnamoyltransferase/*N*-hydroxycinnamoyl/benzoyltransferase/deacetylvindoline 4-*O*-acetyltransferase
CAGR: Compound annual growth rate
CAPE: Caffeic acid phenethyl ester
DMAPP: Dimethylallyl diphosphate
EO: Essential oils
FPP: Farnesyl diphosphate
GFPP: Geranylfarnesyl diphosphate
GGPP: Geranylgeranyl diphosphate
GPP: Geranyl diphosphate
HGA: Homogentisate
IPP: Isopentenyl pyrophosphate
MA: Muconic acid
MEP: Methylerythritol-phosphate
MVA: Mevalonate
NR: Natural rubber
PABA: p-Aminobenzoic acid
*p*CA: *p*-Coumarate
PCA: Protocatechuate
PET: Polyethylene terephthalate
p-HS: p-Hydroxystyrene
PP: Diphosphate
PSPP: Presqualene diphosphate
PVP: Polyvinylphenol
REF: Rubber elongation factor
SA: Salicylic acid
SRPP: Small rubber particle protein
SSU: Small subunit

## References

[1] Rosales-Calderon O, Arantes V (2019). A review on commercial-scale high-value products that can be produced alongside cellulosic ethanol. Biotechnol Biofuels.

[2] Baral NR, Sundstrom ER, Das L, Gladden J, Eudes A, Mortimer JC, Singer SW, Mukhopadhyay A, Scown CD (2019). Approaches for more efficient biological conversion of lignocellulosic feedstocks to biofuels and bioproducts. ACS Sustain Chem Eng.

[3] Amoah J, Kahar P, Ogino C, Kondo A (2019). Bioenergy and biorefinery: feedstock, biotechnological conversion, and products. Biotechnol J.

[4] Bailey-Serres J, Parker JE, Ainsworth EA, Oldroyd GED, Schroeder JI (2019). Genetic strategies for improving crop yields. Nature.

[5] Ralph J, Lapierre C, Boerjan W (2019). Lignin structure and its engineering. Curr Opin Biotechnol.

[6] Farré G, Blancquaert D, Capell T, Straeten DVD, Christou P, Zhu C (2014). Engineering complex metabolic pathways in plants. Annu Rev Plant Biol.

[7] Yuan L, Grotewold E (2015). Metabolic engineering to enhance the value of plants as green factories. Metab Eng.

[8] Yang ML, Baral NR, Simmons BA, Mortimer JC, Shih PM, Scown CD: Accumulation of high-value bioproducts *in planta* can improve the economics of advanced biofuels. In: Proceedings of the National Academy of Sciences of the United States of America **(in press)**. 10.1073/pnas.2000053117 PMC7165473 32220956

[9] Maeda H, Dudareva N (2012). The shikimate pathway and aromatic amino acid biosynthesis in plants. Annu Rev Plant Biol.

[10] Lee J-H, Wendisch VF (2017). Biotechnological production of aromatic compounds of the extended shikimate pathway from renewable biomass. J Biotechnol.

[11] Huccetogullari D, Luo ZW, Lee SY (2019). Metabolic engineering of microorganisms for production of aromatic compounds. Microb Cell Fact.

[12] Takayuki T, Alisdair RF (2017). An overview of compounds derived from the shikimate and phenylpropanoid pathways and their medicinal importance. Mini-Rev Med Chem.

[13] Kawaguchi H, Ogino C, Kondo A (2017). Microbial conversion of biomass into bio-based polymers. Bioresour Technol.

[14] Bai Z, Phuan WC, Ding J, Heng TH, Luo J, Zhu Y (2016). Production of terephthalic acid from lignin-based phenolic acids by a cascade fixed-bed process. ACS Catal.

[15] Beers DE, Ramirez JE (1990). Vectran high-performance fibre. J Textile Inst.

[16] Soni MG, Carabin IG, Burdock GA (2005). Safety assessment of esters of *p*-hydroxybenzoic acid (parabens). Food Chem Toxicol.

[17] Wang S, Bilal M, Hu H, Wang W, Zhang X (2018). 4-Hydroxybenzoic acid—a versatile platform intermediate for value-added compounds. Appl Microbiol Biotechnol.

[18] Rodriguez A, Ersig N, Geiselman GM, Seibel K, Simmons BA, Magnuson JK, Eudes A, Gladden JM (2019). Conversion of depolymerized sugars and aromatics from engineered feedstocks by two oleaginous red yeasts. Bioresour Technol.

[19] Kosa M, Ragauskas AJ (2012). Bioconversion of lignin model compounds with oleaginous *Rhodococci*. Appl Microbiol Biotechnol.

[20] Kumar M, Singhal A, Verma PK, Thakur IS (2017). Production and characterization of polyhydroxyalkanoate from lignin derivatives by *Pandoraea* sp. Istkb. ACS Omega.

[21] Sonoki T, Takahashi K, Sugita H, Hatamura M, Azuma Y, Sato T, Suzuki S, Kamimura N, Masai E (2018). Glucose-free cis, cis-muconic acid production via new metabolic designs corresponding to the heterogeneity of lignin. ACS Sustain Chem Eng.

[22] Johnson CW, Salvachúa D, Rorrer NA, Black BA, Vardon DR, St. John PC, Cleveland NS, Dominick G, Elmore JR, Grundl N (2019). Innovative chemicals and materials from bacterial aromatic catabolic pathways. Joule..

[23] Perez JM, Kontur WS, Alherech M, Coplien J, Karlen SD, Stahl SS, Donohue TJ, Noguera DR (2019). Funneling aromatic products of chemically depolymerized lignin into 2-pyrone-4-6-dicarboxylic acid with *Novosphingobium aromaticivorans*. Green Chem.

[24] Siebert M, Sommer S, Li S, Wang Z, Severin K, Heide L (1996). Genetic engineering of plant secondary metabolism (accumulation of 4-hydroxybenzoate glucosides as a result of the expression of the bacterial ubic gene in tobacco). Plant Physiol.

[25] Mayer MJ, Narbad A, Parr AJ, Parker ML, Walton NJ, Mellon FA, Michael AJ (2001). Rerouting the plant phenylpropanoid pathway by expression of a novel bacterial enoyl-CoA hydratase/lyase enzyme function. Plant Cell.

[26] McQualter RB, Chong BF, Meyer K, Van Dyk DE, O’Shea MG, Walton NJ, Viitanen PV, Brumbley SM (2005). Initial evaluation of sugarcane as a production platform for *p*-hydroxybenzoic acid. Plant Biotechnol J.

[27] Eudes A, George A, Mukerjee P, Kim JS, Pollet B, Benke PI, Yang F, Mitra P, Sun L, Çetinkol ÖP (2012). Biosynthesis and incorporation of side-chain-truncated lignin monomers to reduce lignin polymerization and enhance saccharification. Plant Biotechnol J.

[28] Kim KH, Wang Y, Takada M, Eudes A, Yoo CG, Kim CS, Saddler J (2020). Deep eutectic solvent pretreatment of transgenic biomass with increased C6C1 lignin monomers. Front Plant Sci..

[29] Lu F, Karlen SD, Regner M, Kim H, Ralph SA, Sun R-C, Kuroda K-I, Augustin MA, Mawson R, Sabarez H (2015). Naturally p-hydroxybenzoylated lignins in palms. BioEnergy Res..

[30] Kambourakis S, Draths KM, Frost JW (2000). Synthesis of gallic acid and pyrogallol from glucose: replacing natural product isolation with microbial catalysis. J Am Chem Soc.

[31] Chen Z, Shen X, Wang J, Wang J, Yuan Q, Yan Y (2017). Rational engineering of p-hydroxybenzoate hydroxylase to enable efficient gallic acid synthesis via a novel artificial biosynthetic pathway. Biotechnol Bioeng.

[32] Eudes A, Pereira JH, Yogiswara S, Wang G, Teixeira Benites V, Baidoo EEK, Lee TS, Adams PD, Keasling JD, Loqué D (2016). Exploiting the substrate promiscuity of hydroxycinnamoyl-CoA: shikimate hydroxycinnamoyl transferase to reduce lignin. Plant Cell Physiol.

[33] Tarzia A, Montanaro J, Casiello M, Annese C, Nacci A, Maffezzoli A (2017). Synthesis, curing, and properties of an epoxy resin derived from gallic acid. BioResources.

[34] Lipp A, Ferenc D, Gütz C, Geffe M, Vierengel N, Schollmeyer D, Schäfer HJ, Waldvogel SR, Opatz T (2018). A regio- and diastereoselective anodic aryl–aryl coupling in the biomimetic total synthesis of (−)-thebaine. Angew Chem Int Ed.

[35] Khalil I, Quintens G, Junkers T, Dusselier M (2020). Muconic acid isomers as platform chemicals and monomers in the biobased economy. Green Chem.

[36] Xie N-Z, Liang H, Huang R-B, Xu P (2014). Biotechnological production of muconic acid: current status and future prospects. Biotechnol Adv.

[37] Kruyer NS, Peralta-Yahya P (2017). Metabolic engineering strategies to bio-adipic acid production. Curr Opin Biotechnol.

[38] Eudes A, Berthomieu R, Hao Z, Zhao N, Benites VT, Baidoo EEK, Loqué D (2018). Production of muconic acid in plants. Metab Eng.

[39] Fiege H, Voges HW, Hamamoto T, Umemura S, Iwata T, Miki H, Fujita Y, Buysch HJ, Garbe D, Paulus W (2000). Phenol derivatives. Ullmann’s Encycl Ind Chem..

[40] Kim KH, Dutta T, Sun J, Simmons B, Singh S (2018). Biomass pretreatment using deep eutectic solvents from lignin derived phenols. Green Chem.

[41] Kakkar S, Bais S (2014). A review on protocatechuic acid and its pharmacological potential. ISRN Pharmacol.

[42] Otsuka Y, Nakamura M, Shigehara K, Sugimura K, Masai E, Ohara S, Katayama Y (2006). Efficient production of 2-pyrone 4,6-dicarboxylic acid as a novel polymer-based material from protocatechuate by microbial function. Appl Microbiol Biotechnol.

[43] Okamura-Abe Y, Abe T, Nishimura K, Kawata Y, Sato-Izawa K, Otsuka Y, Nakamura M, Kajita S, Masai E, Sonoki T, Katayama Y (2016). Beta-ketoadipic acid and muconolactone production from a lignin-related aromatic compound through the protocatechuate 3,4-metabolic pathway. J Biosci Bioeng.

[44] Eudes A, Sathitsuksanoh N, Baidoo EEK, George A, Liang Y, Yang F, Singh S, Keasling JD, Simmons BA, Loqué D (2015). Expression of a bacterial 3-dehydroshikimate dehydratase reduces lignin content and improves biomass saccharification efficiency. Plant Biotechnol J.

[45] Wu W, Dutta T, Varman AM, Eudes A, Manalansan B, Loqué D, Singh S (2017). Lignin valorization: two hybrid biochemical routes for the conversion of polymeric lignin into value-added chemicals. Sci Rep.

[46] Konda M, Loqué D, Scown C, Kumar R, Singh S (2016). Towards economically sustainable lignocellulosic biorefineries. Valorization of lignocellulosic biomass in a biorefinery: from logistics to environmental and performance impact.

[47] Lucchini JJ, Bonnaveiro N, Cremieux A, Le Goffic F (1993). Mechanism of bactericidal action of phenethyl alcohol in *Escherichia coli*. Curr Microbiol.

[48] Chen C-S, Chen H-W (2004). Enhanced selectivity and formation of ethylbenzene for acetophenone hydrogenation by adsorbed oxygen on Pd/SiO_2_. Appl Catal A.

[49] Hua D, Xu P (2011). Recent advances in biotechnological production of 2-phenylethanol. Biotechnol Adv.

[50] Costa MA, Marques JV, Dalisay DS, Herman B, Bedgar DL, Davin LB, Lewis NG (2013). Transgenic hybrid poplar for sustainable and scalable production of the commodity/specialty chemical, 2-phenylethanol. PLoS ONE.

[51] Qi G, Wang D, Yu L, Tang X, Chai G, He G, Ma W, Li S, Kong Y, Fu C, Zhou G (2015). Metabolic engineering of 2-phenylethanol pathway producing fragrance chemical and reducing lignin in *Arabidopsis*. Plant Cell Rep.

[52] Günther J, Lackus ND, Schmidt A, Huber M, Stödtler H-J, Reichelt M, Gershenzon J, Köllner TG (2019). Separate pathways contribute to the herbivore-induced formation of 2-phenylethanol in poplar. Plant Physiol.

[53] Kamatou GP, Vermaak I, Viljoen AM (2012). Eugenol—from the remote maluku islands to the international market place: a review of a remarkable and versatile molecule. Molecules.

[54] Bendre RS, Rajput JD, Bagul SD, Karandikar P (2016). Outlooks on medicinal properties of eugenol and its synthetic derivatives. Nat Prod Chem Res.

[55] Overhage J, Steinbüchel A, Priefert H (2003). Highly efficient biotransformation of eugenol to ferulic acid and further conversion to vanillin in recombinant strains of *Escherichia coli*. Appl Environ Microbiol.

[56] Dexter R, Qualley A, Kish CM, Ma CJ, Koeduka T, Nagegowda DA, Dudareva N, Pichersky E, Clark D (2007). Characterization of a petunia acetyltransferase involved in the biosynthesis of the floral volatile isoeugenol. Plant J.

[57] Kim S-J, Vassão DG, Moinuddin SGA, Bedgar DL, Davin LB, Lewis NG (2014). Allyl/propenyl phenol synthases from the creosote bush and engineering production of specialty/commodity chemicals, eugenol/isoeugenol, in Escherichia coli. Arch Biochem Biophys.

[58] Vassão DG, Kim S-J, Milhollan JK, Eichinger D, Davin LB, Lewis NG (2007). A pinoresinol-lariciresinol reductase homologue from the creosote bush (*Larrea tridentata*) catalyzes the efficient in vitro conversion of p-coumaryl/coniferyl alcohol esters into the allylphenols chavicol/eugenol, but not the propenylphenols p-anol/isoeugenol. Arch Biochem Biophys.

[59] Koeduka T, Louie GV, Orlova I, Kish CM, Ibdah M, Wilkerson CG, Bowman ME, Baiga TJ, Noel JP, Dudareva N, Pichersky E (2008). The multiple phenylpropene synthases in both *Clarkia breweri* and *Petunia hybrida* represent two distinct protein lineages. Plant J.

[60] Koeduka T, Suzuki S, Iijima Y, Ohnishi T, Suzuki H, Watanabe B, Shibata D, Umezawa T, Pichersky E, Hiratake J (2013). Enhancement of production of eugenol and its glycosides in transgenic aspen plants via genetic engineering. Biochem Biophys Res Commun.

[61] Lu D, Yuan X, Kim S-J, Marques JV, Chakravarthy PP, Moinuddin SGA, Luchterhand R, Herman B, Davin LB, Lewis NG (2017). Eugenol specialty chemical production in transgenic poplar (*Populus tremula × P. alba*) field trials. Plant Biotechnol J..

[62] Jung D-H, Choi W, Choi K-Y, Jung E, Yun H, Kazlauskas RJ, Kim B-G (2013). Bioconversion of *p*-coumaric acid to *p*-hydroxystyrene using phenolic acid decarboxylase from *B. amyloliquefaciens* in biphasic reaction system. Appl Microbiol Biotechnol..

[63] Paz A, Costa-Trigo I, Tugores F, Míguez M, de la Montaña J, Domínguez JM (2019). Biotransformation of phenolic compounds by *Bacillus aryabhattai*. Bioprocess Biosyst Eng.

[64] Shin S-Y, Han NS, Park Y-C, Kim M-D, Seo J-H (2011). Production of resveratrol from *p*-coumaric acid in recombinant *Saccharomyces cerevisiae* expressing 4-coumarate:coenzyme A ligase and stilbene synthase genes. Enzyme Microb Technol.

[65] Lim CG, Fowler ZL, Hueller T, Schaffer S, Koffas MAG (2011). High-yield resveratrol production in engineered *Escherichia coli*. Appl Environ Microbiol.

[66] Kang L, Li Q, Lin J, Guo L (2015). Biosynthesis of resveratrol in blastospore of the macrofungus *Tremella fuciformis*. Mol Biotechnol.

[67] Vardon DR, Franden MA, Johnson CW, Karp EM, Guarnieri MT, Linger JG, Salm MJ, Strathmann TJ, Beckham GT (2015). Adipic acid production from lignin. Energy Environ Sci.

[68] Johnson CW, Beckham GT (2015). Aromatic catabolic pathway selection for optimal production of pyruvate and lactate from lignin. Metab Eng.

[69] Linger JG, Vardon DR, Guarnieri MT, Karp EM, Hunsinger GB, Franden MA, Johnson CW, Chupka G, Strathmann TJ, Pienkos PT, Beckham GT (2014). Lignin valorization through integrated biological funneling and chemical catalysis. Proc Natl Acad Sci.

[70] Salvachúa D, Rydzak T, Auwae R, De Capite A, Black BA, Bouvier JT, Cleveland NS, Elmore JR, Huenemann JD, Katahira R (2019). Metabolic engineering of *Pseudomonas putida* for increased polyhydroxyalkanoate production from lignin. Microb Biotechnol..

[71] Kaneko T. High‐performance polymers from phenolic biomonomers. In: Mathers RT, Meier MAR, editors. Green polymerization methods. Wiley; 2011. p. 263–290. 10.1002/9783527636167.ch12.

[72] Wang S-Q, Kaneko D, Okajima M, Yasaki K, Tateyama S, Kaneko T (2013). Hyperbranched polycoumarates with photofunctional multiple shape memory. Angew Chem Int Ed.

[73] Hatfield RD, Rancour DM, Marita JM (2017). Grass cell walls: a story of cross-linking. Front Plant Sci.

[74] Nishiyama Y, Yun C-S, Matsuda F, Sasaki T, Saito K, Tozawa Y (2010). Expression of bacterial tyrosine ammonia-lyase creates a novel *p*-coumaric acid pathway in the biosynthesis of phenylpropanoids in *Arabidopsis*. Planta.

[75] Tzin V, Rogachev I, Meir S, Moyal Ben Zvi M, Masci T, Vainstein A, Aharoni A, Galili G (2013). Tomato fruits expressing a bacterial feedback-insensitive 3-deoxy-d-arabino-heptulosonate 7-phosphate synthase of the shikimate pathway possess enhanced levels of multiple specialized metabolites and upgraded aroma. J Exp Bot.

[76] Lopez-Nieves S, Yang Y, Timoneda A, Wang M, Feng T, Smith SA, Brockington SF, Maeda HA (2018). Relaxation of tyrosine pathway regulation underlies the evolution of betalain pigmentation in *Caryophyllales*. New Phytol.

[77] Withers S, Lu F, Kim H, Zhu Y, Ralph J, Wilkerson CG (2012). Identification of grass-specific enzyme that acylates monolignols with *p*-coumarate. J Biol Chem.

[78] Petrik DL, Karlen SD, Cass CL, Padmakshan D, Lu F, Liu S, Le Bris P, Antelme S, Santoro N, Wilkerson CG (2014). *p*-Coumaroyl-CoA:monolignol transferase (PMT) acts specifically in the lignin biosynthetic pathway in *Brachypodium distachyon*. Plant J.

[79] Marita JM, Hatfield RD, Rancour DM, Frost KE (2014). Identification and suppression of the *p*-coumaroyl CoA:hydroxycinnamyl alcohol transferase in *Zea mays* L. Plant J.

[80] Smith RA, Gonzales-Vigil E, Karlen SD, Park J-Y, Lu F, Wilkerson CG, Samuels L, Ralph J, Mansfield SD (2015). Engineering monolignol *p*-coumarate conjugates into poplar and *Arabidopsis* lignins. Plant Physiol.

[81] Sibout R, Le Bris P, Legée F, Cézard L, Renault H, Lapierre C (2016). Structural redesigning Arabidopsis lignins into alkali-soluble lignins through the expression of *p*-coumaroyl-CoA: monolignol transferase PMT. Plant Physiol.

[82] Karlen SD, Fasahati P, Mazaheri M, Serate J, Smith RA, Sirobhushanam S, Chen M, Tymkhin VI, Cass CL, Liu S (2020). Assessing the viability of recovering hydroxycinnamic acids from lignocellulosic biorefinery alkaline pretreatment waste streams. ChemSusChem..

[83] Salgado JM, Rodríguez-Solana R, Curiel JA, de las Rivas B, Muñoz R, Domínguez JM (2014). Bioproduction of 4-vinylphenol from corn cob alkaline hydrolyzate in two-phase extractive fermentation using free or immobilized recombinant *E coli* expressing pad gene. Enzyme Microb Technol..

[84] Rodriguez A, Salvachúa D, Katahira R, Black BA, Cleveland NS, Reed M, Smith H, Baidoo EE, Keasling JD, Simmons BA (2017). Base-catalyzed depolymerization of solid lignin-rich streams enables microbial conversion. ACS Sustain Chem Eng.

[85] Sultan Z, Graça I, Li Y, Lima S, Peeva LG, Kim D, Ebrahim MA, Rinaldi R, Livingston AG (2019). Membrane fractionation of liquors from lignin-first biorefining. Chemsuschem.

[86] Ou SY, Luo YL, Huang CH, Jackson M (2009). Production of coumaric acid from sugarcane bagasse. Innov Food Sci Emerg Technol.

[87] Li G, Jones KC, Eudes A, Pidatala VR, Sun J, Xu F, Zhang C, Wei T, Jain R, Birdseye D (2018). Overexpression of a rice BAHD acyltransferase gene in switchgrass (*Panicum virgatum* L.) enhances saccharification. BMC Biotechnol..

[88] Fonseca AC, Lima MS, Sousa AF, Silvestre AJ, Coelho JFJ, Serra AC (2019). Cinnamic acid derivatives as promising building blocks for advanced polymers: synthesis, properties and applications. Polym Chem.

[89] Di Gioia D, Luziatelli F, Negroni A, Ficca AG, Fava F, Ruzzi M (2011). Metabolic engineering of *Pseudomonas fluorescens* for the production of vanillin from ferulic acid. J Biotechnol.

[90] Furuya T, Miura M, Kuroiwa M, Kino K (2015). High-yield production of vanillin from ferulic acid by a coenzyme-independent decarboxylase/oxygenase two-stage process. New Biotechnol.

[91] Johnson CW, Salvachúa D, Khanna P, Smith H, Peterson DJ, Beckham GT (2016). Enhancing muconic acid production from glucose and lignin-derived aromatic compounds via increased protocatechuate decarboxylase activity. Metab Eng Commun.

[92] Johnson CW, Abraham PE, Linger JG, Khanna P, Hettich RL, Beckham GT (2017). Eliminating a global regulator of carbon catabolite repression enhances the conversion of aromatic lignin monomers to muconate in *Pseudomonas putida* KT2440. Metab Eng Commun.

[93] Stubba D, Lahm G, Geffe M, Runyon JW, Arduengo AJ, Opatz T (2015). Xylochemistry—making natural products entirely from wood. Angew Chem Int Ed.

[94] Zhang P, Tang Y, Li N-G, Zhu Y, Duan J-A (2014). Bioactivity and chemical synthesis of caffeic acid phenethyl ester and its derivatives. Molecules.

[95] Domergue F, Kosma DK (2017). Occurrence and biosynthesis of alkyl hydroxycinnamates in plant lipid barriers. Plants.

[96] Mnich E, Bjarnholt N, Eudes A, Harholt J, Holland C, Jørgensen B, Larsen FH, Liu M, Manat R, Meyer AS (2020). Phenolic cross-links: building and de-constructing the plant cell wall. Nat Prod Rep..

[97] Reinoso FAM, Rencoret J, Gutiérrez A, Milagres AMF, del Río JC, Ferraz A (2018). Fate of p-hydroxycinnamates and structural characteristics of residual hemicelluloses and lignin during alkaline-sulfite chemithermomechanical pretreatment of sugarcane bagasse. Biotechnol Biofuels.

[98] Niggeweg R, Michael AJ, Martin C (2004). Engineering plants with increased levels of the antioxidant chlorogenic acid. Nat Biotechnol.

[99] Cheng A-X, Gou J-Y, Yu X-H, Yang H, Fang X, Chen X-Y, Liu C-J (2013). Characterization and ectopic expression of a *Populus* hydroxyacid hydroxycinnamoyltransferase. Mol Plant.

[100] Tetreault HM, Scully ED, Gries T, Palmer NA, Funnell-Harris DL, Baird L, Seravalli J, Dien BS, Sarath G, Clemente TE, Sattler SE (2018). Overexpression of the *Sorghum bicolor* SbCCoAOMT alters cell wall associated hydroxycinnamoyl groups. PLoS ONE.

[101] Buanafina MMdO, Fescemyer HW, Sharma M, Shearer EA (2016). Functional testing of a PF02458 homologue of putative rice arabinoxylan feruloyl transferase genes in *Brachypodium distachyon*. Planta..

[102] Banerjee G, Chattopadhyay P (2019). Vanillin biotechnology: the perspectives and future. J Sci Food Agric.

[103] Nikafshar S, Zabihi O, Hamidi S, Moradi Y, Barzegar S, Ahmadi M, Naebe M (2017). A renewable bio-based epoxy resin with improved mechanical performance that can compete with DGEBA. RSC Adv.

[104] Zhou J, Zhang H, Deng J, Wu Y (2016). High glass-transition temperature acrylate polymers derived from biomasses, syringaldehyde, and vanillin. Macromol Chem Phys.

[105] Ibrahim MNM, Balakrishnan RS, Shamsudeen S, Bahwani SA, Adam F (2012). A concise review of the natural existence, synthesis, properties, and applications of syringaldehyde. BioResources.

[106] Qian Y, Otsuka Y, Sonoki T, Mukhopadhyay B, Nakamura M, Jellison J, Goodell B (2016). Engineered microbial production of 2-pyrone-4,6-dicarboxylic acid from lignin residues for use as an industrial platform chemical. BioResources..

[107] Kundu A (2017). Vanillin biosynthetic pathways in plants. Planta.

[108] Kim H, Ralph J, Lu F, Ralph SA, Boudet A-M, MacKay JJ, Sederoff RR, Ito T, Kawai S, Ohashi H, Higuchi T (2003). NMR analysis of lignins in CAD-deficient plants. Part 1. Incorporation of hydroxycinnamaldehydes and hydroxybenzaldehydes into lignins. Org Biomol Chem..

[109] Baucher M, Chabbert B, Pilate G, Van Doorsselaere J, Tollier MT, Petit-Conil M, Cornu D, Monties B, Van Montagu M, Inze D (1996). Red xylem and higher lignin extractability by down-regulating a cinnamyl alcohol dehydrogenase in poplar. Plant Physiol.

[110] MacKay JJ, O’Malley DM, Presnell T, Booker FL, Campbell MM, Whetten RW, Sederoff RR (1997). Inheritance, gene expression, and lignin characterization in a mutant pine deficient in cinnamyl alcohol dehydrogenase. Proc Natl Acad Sci.

[111] Kim KH, Eudes A, Jeong K, Yoo CG, Kim CS, Ragauskas A (2019). Integration of renewable deep eutectic solvents with engineered biomass to achieve a closed-loop biorefinery. Proc Natl Acad Sci.

[112] Jacquet N, Eudes A, Dutta T, Kim KH, Bouxin F, Benites V, Baidoo E, Singh S, Simmons B, Loqué D, Richel A (2020). Influence of hydrocracking and ionic liquid pretreatments on composition and properties of *Arabidopsis thaliana* wild type and CAD mutant lignins. Renew Energy.

[113] Rodrigues AE, Pinto PCdOR, Barreiro MF, da Esteves Costa CA, da Ferreira Mota MI, Fernandes I (2018). An integrated approach for added-value products from lignocellulosic biorefineries: vanillin, syringaldehyde, polyphenols and polyurethane.

[114] Anderson NA, Tobimatsu Y, Ciesielski PN, Ximenes E, Ralph J, Donohoe BS, Ladisch M, Chapple C (2015). Manipulation of guaiacyl and syringyl monomer biosynthesis in an *Arabidopsis* cinnamyl alcohol dehydrogenase mutant results in atypical lignin biosynthesis and modified cell wall structure. Plant Cell.

[115] Scully ED, Gries T, Palmer NA, Sarath G, Funnell-Harris DL, Baird L, Twigg P, Seravalli J, Clemente TE, Sattler SE (2018). Overexpression of SbMyb60 in *Sorghum bicolor* impacts both primary and secondary metabolism. New Phytol.

[116] Inoue S, Morita R, Kuwata K, Kunieda T, Ueda H, Hara-Nishimura I, Minami Y (2018). Tissue-specific and intracellular localization of indican synthase from *Polygonum tinctorium*. Plant Physiol Biochem.

[117] Minami Y, Takao H, Kanafuji T, Miura K, Kondo M, Hara-Nishimura I, Nishimura M, Matsubara H (1997). β-Glucosidase in the indigo plant: intracellular localization and tissue specific expression in leaves. Plant Cell Physiol.

[118] Krishnaswamy NR, Sundaresan CN (2012). Fascinating organic molecules from nature. Resonance.

[119] Sharma S, Chandraprabha M (2013). Present status of plant derived indigo dye-A review. Int J Res Eng Technol.

[120] Langholtz M, Stokes B, Eaton L. 2016 Billion-ton report: advancing domestic resources for a thriving bioeconomy. In: Economic availability of feedstock. Oak Ridge National Laboratory, Oak Ridge, Tennessee, managed by UT-Battelle, LLC for the US Department of Energy 2016, 2016:1–411.

[121] Warzecha H, Frank A, Peer M, Gillam EMJ, Guengerich FP, Unger M (2007). Formation of the indigo precursor indican in genetically engineered tobacco plants and cell cultures. Plant Biotechnol J.

[122] Fräbel S, Wagner B, Krischke M, Schmidts V, Thiele CM, Staniek A, Warzecha H (2018). Engineering of new-to-nature halogenated indigo precursors in plants. Metab Eng.

[123] Tzin V, Malitsky S, Zvi MMB, Bedair M, Sumner L, Aharoni A, Galili G (2012). Expression of a bacterial feedback-insensitive 3-deoxy-D-arabino-heptulosonate 7-phosphate synthase of the shikimate pathway in Arabidopsis elucidates potential metabolic bottlenecks between primary and secondary metabolism. New Phytol.

[124] Tozawa Y, Hasegawa H, Terakawa T, Wakasa K (2001). Characterization of rice anthranilate synthase α-subunit genes *OASA1* and *OASA2*. Tryptophan accumulation in transgenic rice expressing a feedback-insensitive mutant of *OASA1*. Plant Physiol..

[125] Maki T, Takeda K (2000). Benzoic acid and derivatives. Ullmann’s Encycl Ind Chem..

[126] von Rymon Lipinski GW, Zorn H, Czermak P (2013). Sweeteners. Biotechnology of food and feed additives.

[127] Hebbar N, Praveen BM, Prasanna BM, Venkatesha TV, Abd Hamid SB (2014). Anthranilic acid as corrosion inhibitor for mild steel in hydrochloric acid media. Procedia Mater Sci.

[128] Graham GG, Parnham MJ (2016). Fenamates. Compendium of inflammatory diseases.

[129] Basset GJC, Quinlivan EP, Ravanel S, Rébeillé F, Nichols BP, Shinozaki K, Seki M, Adams-Phillips LC, Giovannoni JJ, Gregory JF, Hanson AD (2004). Folate synthesis in plants: the *p*-aminobenzoate branch is initiated by a bifunctional PabA-PabB protein that is targeted to plastids. Proc Natl Acad Sci USA.

[130] Niyogi KK, Fink GR (1992). Two anthranilate synthase genes in Arabidopsis: defense-related regulation of the tryptophan pathway. Plant Cell.

[131] Niyogi KK, Last RL, Fink GR, Keith B (1993). Suppressors of trp1 fluorescence identify a new *Arabidopsis* gene, TRP4, encoding the anthranilate synthase beta subunit. Plant Cell.

[132] Last RL, Fink GR (1988). Tryptophan-requiring mutants of the plant *Arabidopsis thaliana*. Science.

[133] Quiel JA, Bender J (2003). Glucose conjugation of anthranilate by the arabidopsis UGT74F2 glucosyltransferase is required for tryptophan mutant blue fluorescence. J Biol Chem.

[134] Loqué D, Eudes A. Modified plants and methods for producing modified lignin by modulating expression of acyltransferases. 2019.

[135] Strobbe S, Van Der Straeten D (2017). Folate biofortification in food crops. Curr Opin Biotechnol.

[136] Eudes A, Bozzo GG, Waller JC, Naponelli V, Lim E-K, Bowles DJ, Gregory JF, Hanson AD (2008). Metabolism of the folate precursor *p*-aminobenzoate in plants: glucose ester formation and vacuolar storage. J Biol Chem.

[137] Christianson DW (2017). Structural and chemical biology of terpenoid cyclases. Chem Rev.

[138] Ashour M, Wink M, Gershenzon J. Biochemistry of terpenoids: monoterpenes, sesquiterpenes and diterpenes. In: Wink M, editor. Annual plant reviews. Biochemistry of plant secondary metabolism. vol. 40. 2010: 258-303.

[139] Tong W-Y, Ramawat KG, Mérillon J-M (2013). Biotransformation of terpenoids and steroids. Natural products: phytochemistry, botany and metabolism of alkaloids, phenolics and terpenes.

[140] Vranová E, Coman D, Gruissem W (2012). Structure and dynamics of the isoprenoid pathway network. Mol Plant.

[141] Webb H, Foley WJ, Külheim C (2015). The genetic basis of foliar terpene yield: Implications for breeding and profitability of Australian essential oil crops. Plant Biotechnol.

[142] Berthelot K, Estevez Y, Deffieux A, Peruch F (2012). Isopentenyl diphosphate isomerase: a checkpoint to isoprenoid biosynthesis. Biochimie.

[143] Nakamura A, Shimada H, Masuda T, Ohta H (2001). Takamiya K-i: Two distinct isopentenyl diphosphate isomerases in cytosol and plastid are differentially induced by environmental stresses in tobacco. FEBS Lett.

[144] Okada K, Kasahara H, Yamaguchi S, Kawaide H, Kamiya Y, Nojiri H, Yamane H (2008). Genetic evidence for the role of isopentenyl diphosphate isomerases in the mevalonate pathway and plant development in *Arabidopsis*. Plant Cell Physiol.

[145] McGarvey DJ, Croteau R (1995). Terpenoid metabolism. Plant Cell.

[146] Rohmer M (1999). The discovery of a mevalonate-independent pathway for isoprenoid biosynthesis in bacteria, algae and higher plants. Nat Prod Rep.

[147] Lange BM, Rujan T, Martin W, Croteau R (2000). Isoprenoid biosynthesis: the evolution of two ancient and distinct pathways across genomes. Proc Natl Acad Sci.

[148] Hoshino Y, Gaucher EA (2018). On the origin of isoprenoid biosynthesis. Mol Biol Evol.

[149] Li M, Hou F, Wu T, Jiang X, Li F, Liu H, Xian M, Zhang H (2020). Recent advances of metabolic engineering strategies in natural isoprenoid production using cell factories. Nat Prod Rep..

[150] Dudareva N, Andersson S, Orlova I, Gatto N, Reichelt M, Rhodes D, Boland W, Gershenzon J (2005). The nonmevalonate pathway supports both monoterpene and sesquiterpene formation in snapdragon flowers. Proc Natl Acad Sci USA.

[151] Rodríguez-Concepción M, Boronat A (2015). Breaking new ground in the regulation of the early steps of plant isoprenoid biosynthesis. Curr Opin Plant Biol.

[152] Zhang Y, Nielsen J, Liu Z (2017). Engineering yeast metabolism for production of terpenoids for use as perfume ingredients, pharmaceuticals and biofuels. FEMS Yeast Res..

[153] Moses T, Pollier J, Thevelein JM, Goossens A (2013). Bioengineering of plant (tri)terpenoids: from metabolic engineering of plants to synthetic biology in vivo and in vitro. New Phytol.

[154] Vickers CE, Bongers M, Liu Q, Delatte T, Bouwmeester H (2014). Metabolic engineering of volatile isoprenoids in plants and microbes. Plant Cell Environ.

[155] Hemmerlin A, Harwood JL, Bach TJ (2012). A raison d’être for two distinct pathways in the early steps of plant isoprenoid biosynthesis?. Prog Lipid Res.

[156] Banerjee A, Sharkey TD (2014). Methylerythritol 4-phosphate (MEP) pathway metabolic regulation. Nat Prod Rep.

[157] Cordoba E, Salmi M, León P (2009). Unravelling the regulatory mechanisms that modulate the MEP pathway in higher plants. J Exp Bot.

[158] Wang Q, Quan S, Xiao H (2019). Towards efficient terpenoid biosynthesis: manipulating IPP and DMAPP supply. Bioresour Bioprocess.

[159] Korth KL, Jaggard DAW, Dixon RA (2000). Developmental and light-regulated post-translational control of 3-hydroxy-3-methylglutaryl-CoA reductase levels in potato. Plant J.

[160] Leivar P, Antolín-Llovera M, Ferrero S, Closa M, Arró M, Ferrer A, Boronat A, Campos N (2011). Multilevel control of *Arabidopsis* 3-hydroxy-3-methylglutaryl coenzyme A reductase by protein phosphatase 2A. Plant Cell.

[161] Friesen JA, Rodwell VW (2004). The 3-hydroxy-3-methylglutaryl coenzyme-A (HMG-CoA) reductases. Genome Biol.

[162] Kiran U, Ram M, Khan MA, Khan S, Jha P, Alam A, Abdin MZ (2010). Structural and functional characterization of HMG-COA reductase from *Artemisia annua*. Bioinformation.

[163] Gershenzon J, Kreis W. Biochemistry of terpenoids: monoterpenes, sesquiterpenes, diterpenes, sterols, cardiac glycosides and steroid saponins. In Annual Plant Reviews online. Roberts JA, editor. 2018. p. 218–94.

[164] Ro D-K, Paradise EM, Ouellet M, Fisher KJ, Newman KL, Ndungu JM, Ho KA, Eachus RA, Ham TS, Kirby J (2006). Production of the antimalarial drug precursor artemisinic acid in engineered yeast. Nature.

[165] Engels B, Dahm P, Jennewein S (2008). Metabolic engineering of taxadiene biosynthesis in yeast as a first step towards Taxol (Paclitaxel) production. Metab Eng.

[166] Lee A-R, Kwon M, Kang M-K, Kim J, Kim S-U, Ro D-K (2019). Increased sesqui- and triterpene production by co-expression of HMG-CoA reductase and biotin carboxyl carrier protein in tobacco (*Nicotiana benthamiana*). Metab Eng.

[167] Kim Y-J, Lee OR, Oh JY, Jang M-G, Yang D-C (2014). Functional analysis of 3-hydroxy-3-methylglutaryl coenzyme A reductase encoding genes in triterpene saponin-producing ginseng. Plant Physiol.

[168] Liao P, Wang H, Hemmerlin A, Nagegowda DA, Bach TJ, Wang M, Chye M-L (2014). Past achievements, current status and future perspectives of studies on 3-hydroxy-3-methylglutaryl-CoA synthase (HMGS) in the mevalonate (MVA) pathway. Plant Cell Rep.

[169] Nagegowda Dinesh A, Bach Thomas J, Chye M-L (2004). *Brassica juncea* 3-hydroxy-3-methylglutaryl (HMG)-CoA synthase 1: expression and characterization of recombinant wild-type and mutant enzymes. Biochem J.

[170] Montamat F, Guilloton M, Karst F, Delrot S (1995). Isolation and characterization of a cDNA encoding Arabidopsis thaliana 3-hydroxy-3-methylglutaryl-coenzyme A synthase. Gene.

[171] Wegener A, Gimbel W, Werner T, Hani J, Ernst D, Sandermann H (1997). Molecular cloning of ozone-inducible protein from *Pinus sylvestris* L. with high sequence similarity to vertebrate 3-hydroxy-3-methylglutaryl-CoA-synthase. Biochim Biophys Acta..

[172] Sirinupong N, Suwanmanee P, Doolittle RF, Suvachitanont W (2005). Molecular cloning of a new cDNA and expression of 3-hydroxy-3-methylglutaryl-CoA synthase gene from *Hevea brasiliensis*. Planta.

[173] Meng X, Song Q, Ye J, Wang L, Xu F (2017). Characterization, function, and transcriptional profiling analysis of 3-hydroxy-3-methylglutaryl-CoA synthase gene (GbHMGS1) towards stresses and exogenous hormone treatments in *Ginkgo biloba*. Molecules.

[174] Zhou M, Zhang Q, Wang C, Chen L, Sun Z, Zhu X, Tang Y, Shao J, Wu Y (2015). Characterization of genes involved in isoprenoid diphosphate biosynthesis in maize. J Plant Growth Regul.

[175] Alex D, Bach TJ, Chye M-L (2000). Expression of *Brassica juncea* 3-hydroxy-3-methylglutaryl CoA synthase is developmentally regulated and stress-responsive. Plant J.

[176] Ren A, Ouyang X, Shi L, Jiang A-L, Mu D-S, Li M-J, Han Q, Zhao M-W (2013). Molecular characterization and expression analysis of GlHMGS, a gene encoding hydroxymethylglutaryl-CoA synthase from *Ganoderma lucidum* (Ling-zhi) in ganoderic acid biosynthesis pathway. World J Microbiol Biotechnol.

[177] Wang H, Nagegowda DA, Rawat R, Bouvier-Navé P, Guo D, Bach TJ, Chye M-L (2012). Overexpression of *Brassica juncea* wild-type and mutant HMG-CoA synthase 1 in *Arabidopsis* up-regulates genes in sterol biosynthesis and enhances sterol production and stress tolerance. Plant Biotechnol J.

[178] Liao P, Chen X, Wang M, Bach TJ, Chye M-L (2018). Improved fruit α-tocopherol, carotenoid, squalene and phytosterol contents through manipulation of *Brassica juncea* 3-hydroxy-3-methylglutaryl-CoA synthase 1 in transgenic tomato. Plant Biotechnol J.

[179] Ghirardo A, Wright LP, Bi Z, Rosenkranz M, Pulido P, Rodríguez-Concepción M, Niinemets Ü, Brüggemann N, Gershenzon J, Schnitzler J-P (2014). Metabolic flux analysis of plastidic isoprenoid biosynthesis in poplar leaves emitting and nonemitting isoprene. Plant Physiol.

[180] Shi J, Fei X, Hu Y, Liu Y, Wei A (2019). Identification of key genes in the synthesis pathway of volatile terpenoids in fruit of *Zanthoxylum Bungeanum* maxim. Forests.

[181] Walter MH, Floss DS, Paetzold H, Manke K, Vollrath J, Brandt W, Strack D, Bach TJ, Rohmer M (2013). Control of plastidial isoprenoid precursor supply: divergent 1-deoxy-d-xylulose 5-phosphate synthase (DXS) isogenes regulate the allocation to primary or secondary metabolism. Isoprenoid synthesis in plants and microorganisms: new concepts and experimental approaches.

[182] Tong Y, Su P, Zhao Y, Zhang M, Wang X, Liu Y, Zhang X, Gao W, Huang L (2015). Molecular cloning and characterization of DXS and DXR genes in the terpenoid biosynthetic pathway of *Tripterygium wilfordii*. Int J Mol Sci.

[183] Estévez JM, Cantero A, Reindl A, Reichler S, León P (2001). 1-deoxy-d-xylulose-5-phosphate synthase, a limiting enzyme for plastidic isoprenoid biosynthesis in plants. J Biol Chem.

[184] Wright LP, Rohwer JM, Ghirardo A, Hammerbacher A, Ortiz-Alcaide M, Raguschke B, Schnitzler J-P, Gershenzon J, Phillips MA (2014). Deoxyxylulose 5-phosphate synthase controls flux through the methylerythritol 4-phosphate pathway in *Arabidopsis*. Plant Physiol.

[185] Pokhilko A, Bou-Torrent J, Pulido P, Rodríguez-Concepción M, Ebenhöh O (2015). Mathematical modelling of the diurnal regulation of the MEP pathway in *Arabidopsis*. New Phytol.

[186] Lois LM, Rodríguez-Concepción M, Gallego F, Campos N, Boronat A (2000). Carotenoid biosynthesis during tomato fruit development: regulatory role of 1-deoxy-d-xylulose 5-phosphate synthase. Plant J.

[187] Enfissi EMA, Fraser PD, Lois L-M, Boronat A, Schuch W, Bramley PM (2005). Metabolic engineering of the mevalonate and non-mevalonate isopentenyl diphosphate-forming pathways for the production of health-promoting isoprenoids in tomato. Plant Biotechnol J.

[188] Gong YF, Liao ZH, Guo BH, Sun XF, Tang KX (2006). Molecular cloning and expression profile analysis of *Ginkgo biloba* DXS gene encoding 1-deoxy-d-xylulose 5-phosphate synthase, the first committed enzyme of the 2-C-methyl-d-erythritol 4-phosphate pathway. Planta Med.

[189] Morris WL, Ducreux LJM, Hedden P, Millam S, Taylor MA (2006). Overexpression of a bacterial 1-deoxy-d-xylulose 5-phosphate synthase gene in potato tubers perturbs the isoprenoid metabolic network: implications for the control of the tuber life cycle. J Exp Bot.

[190] Simpson K, Quiroz LF, Rodriguez-Concepción M, Stange CR (2016). Differential contribution of the first two enzymes of the MEP pathway to the supply of metabolic precursors for carotenoid and chlorophyll biosynthesis in carrot (*Daucus carota*). Front Plant Sci.

[191] Cordoba E, Porta H, Arroyo A, San Román C, Medina L, Rodríguez-Concepción M, León P (2011). Functional characterization of the three genes encoding 1-deoxy-D-xylulose 5-phosphate synthase in maize. J Exp Bot.

[192] Banerjee A, Wu Y, Banerjee R, Li Y, Yan H, Sharkey TD (2013). Feedback inhibition of deoxy-D-xylulose-5-phosphate synthase regulates the methylerythritol 4-phosphate pathway. J Biol Chem.

[193] Carretero-Paulet L, Ahumada I, Cunillera N, Rodríguez-Concepción M, Ferrer A, Boronat A, Campos N (2002). Expression and molecular analysis of the *Arabidopsis DXR* gene encoding 1-deoxy-d-xylulose 5-phosphate reductoisomerase, the first committed enzyme of the 2-C-methyl-d-erythritol 4-phosphate pathway. Plant Physiol.

[194] Carretero-Paulet L, Cairó A, Botella-Pavía P, Besumbes O, Campos N, Boronat A, Rodríguez-Concepción M (2006). Enhanced flux through the methylerythritol 4-phosphate pathway in *Arabidopsis* plants overexpressing deoxyxylulose 5-phosphate reductoisomerase. Plant Mol Biol.

[195] Mahmoud SS, Croteau RB (2001). Metabolic engineering of essential oil yield and composition in mint by altering expression of deoxyxylulose phosphate reductoisomerase and menthofuran synthase. Proc Natl Acad Sci.

[196] Hasunuma T, Takeno S, Hayashi S, Sendai M, Bamba T, Yoshimura S, Tomizawa K-I, Fukusaki E, Miyake C (2008). Overexpression of 1-deoxy-d-xylulose-5-phosphate reductoisomerase gene in chloroplast contributes to increment of isoprenoid production. J Biosci Bioeng.

[197] Campbell R, Freitag S, Bryan GJ, Stewart D, Taylor MA (2016). Environmental and genetic factors associated with solanesol accumulation in potato leaves. Front Plant Sci..

[198] Hans J, Hause B, Strack D, Walter MH (2004). Cloning, characterization, and immunolocalization of a mycorrhiza-inducible 1-deoxy-d-xylulose 5-phosphate reductoisomerase in arbuscule-containing cells of maize. Plant Physiol.

[199] Walter MH, Fester T, Strack D (2000). Arbuscular mycorrhizal fungi induce the non-mevalonate methylerythritol phosphate pathway of isoprenoid biosynthesis correlated with accumulation of the ‘yellow pigment’ and other apocarotenoids. Plant J.

[200] Tatsis EC, O’Connor SE (2016). New developments in engineering plant metabolic pathways. Curr Opin Biotechnol.

[201] Lange BM, Ahkami A (2013). Metabolic engineering of plant monoterpenes, sesquiterpenes and diterpenes—current status and future opportunities. Plant Biotechnol J.

[202] Wright RC, Nemhauser J (2019). Plant synthetic biology: quantifying the “known unknowns” and discovering the “unknown unknowns”. Plant Physiol.

[203] Castillo C, Pereira V, Abuelo A, Hernandez J (2013). Effect of supplementation with antioxidants on the quality of bovine milk and meat production. Sci World J.

[204] Tetali SD (2019). Terpenes and isoprenoids: a wealth of compounds for global use. Planta.

[205] George KW, Alonso-Gutierrez J, Keasling JD, Lee TS, Schrader J, Bohlmann J (2015). Isoprenoid drugs, biofuels, and chemicals—artemisinin, farnesene, and beyond. Biotechnology of isoprenoids.

[206] Nogueira M, Enfissi EMA, Almeida J, Fraser PD (2018). Creating plant molecular factories for industrial and nutritional isoprenoid production. Curr Opin Biotechnol.

[207] Kainer D, Padovan A, Degenhardt J, Krause S, Mondal P, Foley WJ, Külheim C (2019). High marker density GWAS provides novel insights into the genomic architecture of terpene oil yield in *Eucalyptus*. New Phytol.

[208] Papa G, Kirby J, Murthy Konda NVSN, Tran K, Singh S, Keasling JD, Peter GF, Simmons BA (2017). Development of an integrated approach for α-pinene recovery and sugar production from loblolly pine using ionic liquids. Green Chem.

[209] Zhang Q-W, Lin L-G, Ye W-C (2018). Techniques for extraction and isolation of natural products: a comprehensive review. Chin Med.

[210] Birtić S, Dussort P, Pierre F-X, Bily AC, Roller M (2015). Carnosic acid. Phytochemistry.

[211] Grace MH, Qiang Y, Sang S, Lila MA (2017). One-step isolation of carnosic acid and carnosol from rosemary by centrifugal partition chromatography. J Sep Sci.

[212] Zi J, Peters RJ (2013). Characterization of CYP76AH4 clarifies phenolic diterpenoid biosynthesis in the Lamiaceae. Org Biomol Chem.

[213] Ignea C, Athanasakoglou A, Ioannou E, Georgantea P, Trikka FA, Loupassaki S, Roussis V, Makris AM, Kampranis SC (2016). Carnosic acid biosynthesis elucidated by a synthetic biology platform. Proc Natl Acad Sci.

[214] Zhang C. Biosynthesis of carotenoids and apocarotenoids by microorganisms and their industrial potential. In: Progress in carotenoid research. 2018: 85–105.

[215] Anunciato TP, da Rocha Filho PA (2012). Carotenoids and polyphenols in nutricosmetics, nutraceuticals, and cosmeceuticals. J Cosm Dermatol.

[216] Eggersdorfer M, Wyss A (2018). Carotenoids in human nutrition and health. Arch Biochem Biophys.

[217] Nagarajan J, Ramanan RN, Raghunandan ME, Galanakis CM, Krishnamurthy NP, Galanakis CM (2017). Carotenoids. Nutraceutical and functional food components.

[218] Barredo JL, García-Estrada C, Kosalkova K, Barreiro C (2017). Biosynthesis of astaxanthin as a main carotenoid in the heterobasidiomycetous yeast *Xanthophyllomyces dendrorhous*. J Fungi.

[219] Davinelli S, Nielsen ME, Scapagnini G (2018). Astaxanthin in skin health, repair, and disease: a comprehensive review. Nutrients.

[220] Zhong Y-J, Huang J-C, Liu J, Li Y, Jiang Y, Xu Z-F, Sandmann G, Chen F (2011). Functional characterization of various algal carotenoid ketolases reveals that ketolating zeaxanthin efficiently is essential for high production of astaxanthin in transgenic *Arabidopsis*. J Exp Bot.

[221] Yu L, Chen Q, Peng Y, Xie L, Liu D, Han M, Chen F, Xiao S, Huang J, Li J (2019). *Arabidopsis thaliana* plants engineered to produce astaxanthin show enhanced oxidative stress tolerance and bacterial pathogen resistance. J Agric Food Chem..

[222] Sathasivam R, Ki J-S (2018). A review of the biological activities of microalgal carotenoids and their potential use in healthcare and cosmetic industries. Mar Drugs.

[223] Moise AR, Al-Babili S, Wurtzel ET (2014). Mechanistic aspects of carotenoid biosynthesis. Chem Rev.

[224] Cunningham FX, Gantt E (2001). One ring or two? Determination of ring number in carotenoids by lycopene epsilon-cyclases. Proc Natl Acad Sci USA.

[225] Zhu Q, Zeng D, Yu S, Cui C, Li J, Li H, Chen J, Zhang R, Zhao X, Chen L, Liu Y-G (2018). From Golden rice to aSTARice: bioengineering astaxanthin biosynthesis in rice endosperm. Mol Plant.

[226] Ha S-H, Kim JK, Jeong YS, You M-K, Lim S-H, Kim J-K (2019). Stepwise pathway engineering to the biosynthesis of zeaxanthin, astaxanthin and capsanthin in rice endosperm. Metab Eng.

[227] Breitenbach J, Nogueira M, Farré G, Zhu C, Capell T, Christou P, Fleck G, Focken U, Fraser PD, Sandmann G (2016). Engineered maize as a source of astaxanthin: processing and application as fish feed. Trans Res.

[228] Jan S, Abbas N, Jan S, Abbas N (2018). Chemistry of Himalayan phytochemicals. Himalayan phytochemicals.

[229] Mène-Saffrané L (2017). Vitamin E biosynthesis and its regulation in plants. Antioxidants.

[230] Sen CK, Khanna S, Roy S (2006). Tocotrienols: vitamin E beyond tocopherols. Life Sci.

[231] Fritsche S, Wang X, Jung C (2017). Recent advances in our understanding of tocopherol biosynthesis in plants: an overview of key genes, functions, and breeding of vitamin E improved crops. Antioxidants.

[232] Jiang Q, Christen S, Shigenaga MK, Ames BN (2001). γ-Tocopherol, the major form of vitamin E in the US diet, deserves more attention. Am J Clin Nutr.

[233] Rizvi S, Raza ST, Ahmed F, Ahmad A, Abbas S, Mahdi F (2014). The role of vitamin E in human health and some diseases. Sultan Qaboos Univ Med J.

[234] Ajjawi I, Shintani D (2004). Engineered plants with elevated vitamin E: a nutraceutical success story. Trends Biotechnol.

[235] Gutbrod K, Romer J, Dörmann P (2019). Phytol metabolism in plants. Prog Lipid Res.

[236] Kimura E, Abe T, Murata K, Kimura T, Otoki Y, Yoshida T, Miyazawa T, Nakagawa K (1870). Identification of OsGGR2, a second geranylgeranyl reductase involved in α-tocopherol synthesis in rice. Sci Rep.

[237] Georgiadou EC, Ntourou T, Goulas V, Manganaris GA, Kalaitzis P, Fotopoulos V (2015). Temporal analysis reveals a key role for VTE5 in vitamin E biosynthesis in olive fruit during on-tree development. Front Plant Sci..

[238] Pellaud S, Mène-Saffrané L (2017). Metabolic origins and transport of vitamin E biosynthetic precursors. Front Plant Sci.

[239] Cahoon EB, Hall SE, Ripp KG, Ganzke TS, Hitz WD, Coughlan SJ (2003). Metabolic redesign of vitamin E biosynthesis in plants for tocotrienol production and increased antioxidant content. Nat Biotechnol.

[240] Che P, Zhao Z-Y, Glassman K, Dolde D, Hu TX, Jones TJ, Gruis DF, Obukosia S, Wambugu F, Albertsen MC (2016). Elevated vitamin E content improves all *trans* β-carotene accumulation and stability in biofortified sorghum. Proc Natl Acad Sci.

[241] Stacey MG, Cahoon RE, Nguyen HT, Cui Y, Sato S, Nguyen CT, Phoka N, Clark KM, Liang Y, Forrester J (2016). Identification of homogentisate dioxygenase as a target for vitamin E biofortification in oilseeds. Plant Physiol.

[242] Global flavor and fragrance market report 2019. https://www.globenewswire.com/news-release/2019/07/02/1877494/0/en/Global-Flavor-and-Fragrance-Market-Report-2019-Growing-Awareness-Among-Customers-to-Buy-Products-that-Contain-Natural-Ingredients.html.

[243] Eslahi H, Fahimi N, Sardarian AR. Chemical composition of essential oils. In: Essential oils in food processing: chemistry, safety and applications. 2017. p. 119–71.

[244] Aprotosoaie AC, Gille E, Trifan A, Luca VS, Miron A (2017). Essential oils of *Lavandula* genus: a systematic review of their chemistry. Phytochem Rev.

[245] Clarke S, Clarke S (2008). Composition of essential oils and other materials. *Essential Chemistry for Aromatherapy (Second Edition)*.

[246] Schempp FM, Drummond L, Buchhaupt M, Schrader J (2018). Microbial cell factories for the production of terpenoid flavor and fragrance compounds. J Agric Food Chem.

[247] Ventura SPM, Silva EFA, Quental MV, Mondal D, Freire MG, Coutinho JAP (2017). Ionic-liquid-mediated extraction and separation processes for bioactive compounds: Past, present, and future trends. Chem Rev.

[248] Chacón MG, Marriott A, Kendrick EG, Styles MQ, Leak DJ (2019). Esterification of geraniol as a strategy for increasing product titre and specificity in engineered *Escherichia coli*. Microb Cell Fact.

[249] Chen W, Viljoen AM (2010). Geraniol—a review of a commercially important fragrance material. S Afr J Bot.

[250] Kutyna DR, Borneman AR (2018). Heterologous production of flavour and aroma compounds in *Saccharomyces cerevisiae*. Genes.

[251] Iijima Y, Gang DR, Fridman E, Lewinsohn E, Pichersky E (2004). Characterization of geraniol synthase from the peltate glands of sweet basil. Plant Physiol.

[252] Yang T, Li J, Wang H-X, Zeng Y (2005). A geraniol-synthase gene from *Cinnamomum tenuipilum*. Phytochemistry.

[253] Li X, Xu Y, Shen S, Yin X, Klee H, Zhang B, Chen K, Hancock R (2017). Transcription factor CitERF71 activates the terpene synthase gene CitTPS16 involved in the synthesis of *E*-geraniol in sweet orange fruit. J Exp Bot.

[254] Dong L, Miettinen K, Goedbloed M, Verstappen FWA, Voster A, Jongsma MA, Memelink J (2013). Krol Svd, Bouwmeester HJ: Characterization of two geraniol synthases from *Valeriana officinalis* and *Lippia dulcis*: Similar activity but difference in subcellular localization. Metab Eng.

[255] Vasilev N, Schmitz C, Grömping U, Fischer R, Schillberg S (2014). Assessment of cultivation factors that affect biomass and geraniol production in transgenic tobacco cell suspension cultures. PLoS ONE.

[256] Masakapalli SK, Ritala A, Dong L, van der Krol AR, Oksman-Caldentey K-M, Ratcliffe RG, Sweetlove LJ (2014). Metabolic flux phenotype of tobacco hairy roots engineered for increased geraniol production. Phytochemistry.

[257] Ritala A, Dong L, Imseng N, Seppänen-Laakso T, Vasilev N, van der Krol S, Rischer H, Maaheimo H, Virkki A, Brändli J (2014). Evaluation of tobacco (*Nicotiana tabacum* L. cv. Petit Havana SR1) hairy roots for the production of geraniol, the first committed step in terpenoid indole alkaloid pathway. J Biotechnol..

[258] Gutensohn M, Orlova I, Nguyen TTH, Davidovich-Rikanati R, Ferruzzi MG, Sitrit Y, Lewinsohn E, Pichersky E, Dudareva N (2013). Cytosolic monoterpene biosynthesis is supported by plastid-generated geranyl diphosphate substrate in transgenic tomato fruits. Plant J.

[259] Kumar SR, Shilpashree HB, Nagegowda DA (2018). Terpene moiety enhancement by overexpression of geranyl(geranyl) diphosphate synthase and geraniol synthase elevates monomeric and dimeric monoterpene indole alkaloids in transgenic *Catharanthus roseus*. Front Plant Sci..

[260] Saiman MZ, Miettinen K, Mustafa NR, Choi YH, Verpoorte R, Schulte AE (2018). Metabolic alteration of *Catharanthus roseus* cell suspension cultures overexpressing geraniol synthase in the plastids or cytosol. Plant Cell Tissue Organ Cult..

[261] Dong L, Jongedijk E, Bouwmeester H, Van Der Krol A (2016). Monoterpene biosynthesis potential of plant subcellular compartments. New Phytol.

[262] Yauk Y-K, Ged C, Wang MY, Matich AJ, Tessarotto L, Cooney JM, Chervin C, Atkinson RG (2014). Manipulation of flavour and aroma compound sequestration and release using a glycosyltransferase with specificity for terpene alcohols. Plant J.

[263] Tamogami S, Agrawal GK, Rakwal R (2016). Methyl jasmonate elicits the biotransformation of geraniol stored as its glucose conjugate into methyl geranate in *Achyranthes bidentata* plant. Plant Physiol Biochem.

[264] Buleandra M, Oprea E, Popa DE, David IG, Moldovan Z, Mihai I, Badea IA (2016). Comparative chemical analysis of *Mentha piperita* and *M. spicata* and a fast assessment of commercial peppermint teas. Nat Prod commun..

[265] Schwab W, Davidovich-Rikanati R, Lewinsohn E (2008). Biosynthesis of plant-derived flavor compounds. Plant J.

[266] Global menthol market insights, forecast to 2025. https://www.wiseguyreports.com/reports/3962281-global-menthol-market-insights-forecast-to-2025.

[267] Banerjee A, Hamberger B (2018). P450s controlling metabolic bifurcations in plant terpene specialized metabolism. Phytochem Rev.

[268] Kirby J, Keasling JD (2009). Biosynthesis of plant isoprenoids: perspectives for microbial engineering. Annu Rev Plant Biol.

[269] Ringer KL, McConkey ME, Davis EM, Rushing GW, Croteau R (2003). Monoterpene double-bond reductases of the (−)-menthol biosynthetic pathway: isolation and characterization of cDNAs encoding (−)-isopiperitenone reductase and (+)-pulegone reductase of peppermint. Arch Biochem Biophys.

[270] Davis EM, Ringer KL, McConkey ME, Croteau R (2005). Monoterpene metabolism Cloning, expression, and characterization of menthone reductases from peppermint. Plant Physiol..

[271] Wildung MR, Croteau RB (2005). Genetic engineering of peppermint for improved essential oil composition and yield. Trans Res.

[272] Currin A, Dunstan MS, Johannissen LO, Hollywood KA, Vinaixa M, Jervis AJ, Swainston N, Rattray NJW, Gardiner JM, Kell DB (2018). Engineering the “missing link” in biosynthetic (−)-menthol production: bacterial isopulegone isomerase. ACS Catal.

[273] Mewalal R, Rai DK, Kainer D, Chen F, Külheim C, Peter GF, Tuskan GA (2017). Plant-derived terpenes: a feedstock for specialty biofuels. Trends Biotechnol.

[274] Peralta-Yahya PP, Zhang F, del Cardayre SB, Keasling JD (2012). Microbial engineering for the production of advanced biofuels. Nature.

[275] Liu C-L, Tian T, Alonso-Gutierrez J, Garabedian B, Wang S, Baidoo EEK, Benites V, Chen Y, Petzold CJ, Adams PD (2018). Renewable production of high density jet fuel precursor sesquiterpenes from *Escherichia coli*. Biotechnol Biofuels.

[276] González-Mas MC, Rambla JL, López-Gresa MP, Blázquez MA, Granell A (2019). Volatile compounds in *Citrus* essential oils: a comprehensive review. Front Plant Sci.

[277] Golmohammadi M, Borghei A, Zenouzi A, Ashrafi N, Taherzadeh MJ (2018). Optimization of essential oil extraction from orange peels using steam explosion. Heliyon.

[278] Karp F, Mihaliak CA, Harris JL, Croteau R (1990). Monoterpene biosynthesis: specificity of the hydroxylations of (−)-limonene by enzyme preparations from peppermint (*Mentha piperita*), spearmint (*Mentha spicata*), and perilla (*Perilla frutescens*) leaves. Arch Biochem Biophys.

[279] Davis EM, Liu H-W, Mander L (2010). Advances in the enzymology of monoterpene cyclization reactions. Comprehensive natural products II.

[280] Morehouse BR, Kumar RP, Matos JO, Olsen SN, Entova S, Oprian DD (2017). Functional and structural characterization of a (+)-limonene synthase from *Citrus sinensis*. Biochemistry.

[281] Krasnyanski S, May RA, Loskutov A, Ball TM, Sink KC (1999). Transformation of the limonene synthase gene into peppermint (*Mentha piperita* L.) and preliminary studies on the essential oil profiles of single transgenic plants. Theor Appl Genet..

[282] Muñoz-Bertomeu J, Ros R, Arrillaga I, Segura J (2008). Expression of spearmint limonene synthase in transgenic spike lavender results in an altered monoterpene composition in developing leaves. Metab Eng.

[283] Ohara K, Ujihara T, Endo T, Sato F, Yazaki K (2003). Limonene production in tobacco with *Perilla* limonene synthase cDNA. J Exp Bot.

[284] Ohara K, Matsunaga E, Nanto K, Yamamoto K, Sasaki K, Ebinuma H, Yazaki K (2010). Monoterpene engineering in a woody plant *Eucalyptus camaldulensis* using a limonene synthase cDNA. Plant Biotechnol J.

[285] Wu S, Schalk M, Clark A, Miles RB, Coates R, Chappell J (2006). Redirection of cytosolic or plastidic isoprenoid precursors elevates terpene production in plants. Nat Biotechnol.

[286] Borghi M, Xie D-Y (2016). Tissue-specific production of limonene in *Camelina sativa* with the *Arabidopsis* promoters of genes BANYULS and FRUITFULL. Planta.

[287] Çitoğlu GS, Acıkara ÖB: Column chromatography for terpenoids and flavonoids. 2012.

[288] Yeo SK, Ali AY, Hayward OA, Turnham D, Jackson T, Bowen ID, Clarkson R (2016). β-bisabolene, a sesquiterpene from the essential oil extract of opoponax (*Commiphora guidottii*), exhibits cytotoxicity in breast cancer cell lines. Phytother Res.

[289] Peralta-Yahya PP, Ouellet M, Chan R, Mukhopadhyay A, Keasling JD, Lee TS (2011). Identification and microbial production of a terpene-based advanced biofuel. Nat Commun.

[290] Davidovich-Rikanati R, Lewinsohn E, Bar E, Iijima Y, Pichersky E, Sitrit Y (2008). Overexpression of the lemon basil α-zingiberene synthase gene increases both mono- and sesquiterpene contents in tomato fruit. Plant J.

[291] Jiang Z, Kempinski C, Bush CJ, Nybo SE, Chappell J (2016). Engineering triterpene and methylated triterpene production in plants provides biochemical and physiological insights into terpene metabolism. Plant Physiol.

[292] Niehaus TD, Okada S, Devarenne TP, Watt DS, Sviripa V, Chappell J (2011). Identification of unique mechanisms for triterpene biosynthesis in *Botryococcus braunii*. Proc Natl Acad Sci USA.

[293] Hillen LW, Pollard G, Wake LV, White N (1982). Hydrocracking of the oils of *Botryococcus braunii* to transport fuels. Biotechnol Bioeng.

[294] Niehaus TD, Kinison S, Okada S, Yeo Y-S, Bell SA, Cui P, Devarenne TP, Chappell J (2012). Functional identification of triterpene methyltransferases from *Botryococcus braunii* race B. J Biol Chem.

[295] Weiss TL, Chun HJ, Okada S, Vitha S, Holzenburg A, Laane J, Devarenne TP (2010). Raman spectroscopy analysis of botryococcene hydrocarbons from the green microalga *Botryococcus braunii*. J Biol Chem.

[296] Kempinski C, Jiang Z, Zinck G, Sato SJ, Ge Z, Clemente TE, Chappell J (2019). Engineering linear, branched-chain triterpene metabolism in monocots. Plant Biotechnol J.

[297] Lozoya-Gloria E, Morales-de la Cruz X, Ozawa-Uyeda TA (2019). The colonial microalgae *Botryococcus braunii* as biorefinery. Microalgae-from physiology to application.

[298] Zhu Y, Romain C, Williams CK (2016). Sustainable polymers from renewable resources. Nature.

[299] Winnacker M, Rieger B (2015). Recent progress in sustainable polymers obtained from cyclic terpenes: Synthesis, properties, and application potential. Chemsuschem.

[300] Bauer N, Brunke J, Kali G (2017). Controlled radical polymerization of myrcene in bulk: mapping the effect of conditions on the system. ACS Sustain Chem Eng.

[301] Cornish K (2017). Alternative natural rubber crops: why should we care?. Technol Innov.

[302] Cornish K (2001). Similarities and differences in rubber biochemistry among plant species. Phytochemistry.

[303] Yip E, Cacioli P (2002). The manufacture of gloves from natural rubber latex. J Allergy Clin Immunol.

[304] Seetang-Nun Y, Sharkey TD, Suvachittanont W (2008). Molecular cloning and characterization of two cDNAs encoding 1-deoxy-d-xylulose 5-phosphate reductoisomerase from *Hevea brasiliensis*. J Plant Physiol.

[305] Ko J-H, Chow K-S, Han K-H (2003). Transcriptome analysis reveals novel features of the molecular events occurring in the laticifers of *Hevea brasiliensis* (para rubber tree). Plant Mol Biol.

[306] Chow K-S, Mat-Isa M-N, Bahari A, Ghazali A-K, Alias H, Mohd-Zainuddin Z, Hoh C-C, Wan K-L (2012). Metabolic routes affecting rubber biosynthesis in *Hevea brasiliensis* latex. J Exp Bot.

[307] Takahashi S, Lee H-J, Yamashita S, Koyama T (2012). Characterization of *cis*-prenyltransferases from the rubber producing plant *Hevea brasiliensis* heterologously expressed in yeast and plant cells. Plant Biotechnol.

[308] Men X, Wang F, Chen G-Q, Zhang H-B, Xian M (2018). Biosynthesis of natural rubber: current state and perspectives. Int J Mol Sci.

[309] Lakusta AM, Kwon M, Kwon E-JG, Stonebloom S, Scheller HV, Ro D-K (2019). Molecular studies of the protein complexes involving *cis*-prenyltransferase in guayule (*Parthenium argentatum*), an alternative rubber-producing plant. Front Plant Sci..

[310] Tang C, Yang M, Fang Y, Luo Y, Gao S, Xiao X, An Z, Zhou B, Zhang B, Tan X (2016). The rubber tree genome reveals new insights into rubber production and species adaptation. Nat Plants.

[311] Cherian S, Ryu SB, Cornish K (2019). Natural rubber biosynthesis in plants, the rubber transferase complex, and metabolic engineering progress and prospects. Plant Biotechnol J.

[312] Veatch ME, Ray DT, Mau CJD, Cornish K (2005). Growth, rubber, and resin evaluation of two-year-old transgenic guayule. Ind Crops Prod.

[313] Venkatachalam P, Priya P, Jayashree R, Rekha K, Thulaseedharan A (2009). Molecular cloning and characterization of a 3-hydroxy-3-methylglutaryl-coenzyme A reductase 1 (hmgr1) gene from rubber tree (*Hevea brasiliensis* Muell. Arg.): a key gene involved in isoprenoid biosynthesis. Physiol Mol Biol Plants..

[314] Jayashree R, Nazeem PA, Rekha K, Sreelatha S, Thulaseedharan A, Krishnakumar R, Kala RG, Vineetha M, Leda P, Jinu U, Venkatachalam P (2018). Over-expression of 3-hydroxy-3-methylglutaryl-coenzyme A reductase 1 (hmgr1) gene under super-promoter for enhanced latex biosynthesis in rubber tree (*Hevea brasiliensis* Muell. Arg.). Plant Physiol Biochem..

[315] Iaffaldano B, Zhang Y, Cornish K (2016). CRISPR/Cas9 genome editing of rubber producing dandelion *Taraxacum kok*-*saghyz* using *Agrobacterium rhizogenes* without selection. Ind Crops Prod.

[316] Shih PM, Liang Y, Loqué D (2016). Biotechnology and synthetic biology approaches for metabolic engineering of bioenergy crops. Plant J.

[317] Altpeter F, Springer NM, Bartley LE, Blechl AE, Brutnell TP, Citovsky V, Conrad LJ, Gelvin SB, Jackson DP, Kausch AP (2016). Advancing crop transformation in the era of genome editing. Plant Cell.

[318] Dobrogojski J, Spychalski M, Luciński R, Borek S (2018). Transgenic plants as a source of polyhydroxyalkanoates. Acta Physiol Plant.

[319] Sadre R, Kuo P, Chen J, Yang Y, Banerjee A, Benning C, Hamberger B (2019). Cytosolic lipid droplets as engineered organelles for production and accumulation of terpenoid biomaterials in leaves. Nat Commun.

[320] Somleva MN, Peoples OP, Snell KD (2013). PHA bioplastics, biochemicals, and energy from crops. Plant Biotechnol J.

[321] Attard TM, Theeuwes E, Gomez LD, Johansson E, Dimitriou I, Wright PC, Clark JH, McQueen-Mason SJ, Hunt AJ (2015). Supercritical extraction as an effective first-step in a maize stover biorefinery. RSC Adv.

[322] Gu T, Held MA, Faik A (2013). Supercritical CO_2_ and ionic liquids for the pretreatment of lignocellulosic biomass in bioethanol production. Environ Technol.

[323] Sundstrom E, Yaegashi J, Yan J, Masson F, Papa G, Rodriguez A, Mirsiaghi M, Liang L, He Q, Tanjore D (2018). Demonstrating a separation-free process coupling ionic liquid pretreatment, saccharification, and fermentation with *Rhodosporidium toruloides* to produce advanced biofuels. Green Chem.

[324] Dong J, Chen Y, Benites VT, Baidoo EEK, Petzold CJ, Beller HR, Eudes A, Scheller HV, Adams PD, Mukhopadhyay A (2019). Methyl ketone production by *Pseudomonas putida* is enhanced by plant-derived amino acids. Biotechnol Bioeng.

